# Marine sulfate-reducing bacteria cause serious corrosion of iron under electroconductive biogenic mineral crust

**DOI:** 10.1111/j.1462-2920.2012.02778.x

**Published:** 2012-07

**Authors:** Dennis Enning, Hendrik Venzlaff, Julia Garrelfs, Hang T Dinh, Volker Meyer, Karl Mayrhofer, Achim W Hassel, Martin Stratmann, Friedrich Widdel

**Affiliations:** 1 Max Planck Institute for Marine MicrobiologyCelsiusstraße 1, D-28359 Bremen, Germany; 2 Max Planck Institute for Iron ResearchMax-Planck-Straße 1, D-40237 Düsseldorf, Germany; 3 Institute for Chemical Technology of Inorganic Materials, Johannes Kepler UniversityAltenberger Straße 69, A-4040 Linz, Austria

## Abstract

Iron (Fe^0^) corrosion in anoxic environments (e.g. inside pipelines), a process entailing considerable economic costs, is largely influenced by microorganisms, in particular sulfate-reducing bacteria (SRB). The process is characterized by formation of black crusts and metal pitting. The mechanism is usually explained by the corrosiveness of formed H_2_S, and scavenge of ‘cathodic’ H_2_ from chemical reaction of Fe^0^ with H_2_O. Here we studied peculiar marine SRB that grew lithotrophically with metallic iron as the only electron donor. They degraded up to 72% of iron coupons (10 mm × 10 mm × 1 mm) within five months, which is a technologically highly relevant corrosion rate (0.7 mm Fe^0^ year^−1^), while conventional H_2_-scavenging control strains were not corrosive. The black, hard mineral crust (FeS, FeCO_3_, Mg/CaCO_3_) deposited on the corroding metal exhibited electrical conductivity (50 S m^−1^). This was sufficient to explain the corrosion rate by electron flow from the metal (4Fe^0^ → 4Fe^2+^ + 8e^−^) through semiconductive sulfides to the crust-colonizing cells reducing sulfate (8e^−^ + SO_4_^2−^ + 9H^+^ → HS^−^ + 4H_2_O). Hence, anaerobic microbial iron corrosion obviously bypasses H_2_ rather than depends on it. SRB with such corrosive potential were revealed at naturally high numbers at a coastal marine sediment site. Iron coupons buried there were corroded and covered by the characteristic mineral crust. It is speculated that anaerobic biocorrosion is due to the promiscuous use of an ecophysiologically relevant catabolic trait for uptake of external electrons from abiotic or biotic sources in sediments.

## Introduction

Iron, the fourth most abundant element in the earth's crust, is the principal redox-active metal in metabolic processes of essentially all living organisms. It is either involved in catalytic quantities as a component of a vast number of proteins, or in much higher, substrate quantities as the external electron donor or acceptor for specially adapted environmental microorganisms referred to as ‘iron bacteria’ (ferrotrophic bacteria, aerobic or anaerobic) or ‘iron-respiring bacteria’ respectively. In most biological functions, iron has the +II (ferrous) or +III (ferric) oxidation state. From a physiological point of view it appears astounding that also the native, metallic element (Fe^0^) can be involved in a biological process; this is anaerobic microbial corrosion. In technology, the process is often referred to as microbially influenced corrosion (MIC).

Iron is the technologically most widely employed metal, due to the abundance of its ores, straightforward melting and excellent mechanical properties. It is globally produced at a 25-fold higher extent (9.3 × 10^8^ t year^−1^) than the second most widely employed metal, aluminum (US Geological Survey, 2011; data for 2009). Iron corrosion including MIC is thus of significant economic relevance. MIC affects industrial water-bearing systems such as oil and gas pipelines (Hamilton, 1985; Li *et al*., 2000; Schwermer *et al*., 2008). It therefore causes, besides economic losses, also failures that are of environmental concern or even hazardous (Duncan *et al*., 2009; Sherar *et al*., 2011). A critical feature of MIC is that it is not as visible as the commonly known rusting of iron under air, but usually occurs as a ‘hidden’ process in the interior of iron pipes or on iron constructions buried in aqueous underground. There is much agreement that sulfate-reducing bacteria (SRB; more generally also sulfate-reducing microorganisms, SRM) are the main culprits of MIC (Hamilton, 1985;Lee *et al*., 1995). Yet, the underlying mechanisms are apparently complex and insufficiently understood (Beech and Sunner, 2007). Their understanding is expected to contribute to the future development of effective mitigation strategies or causative counter measures.

The principal chemical feature in all models of MIC is that iron as a base metal easily gives off electrons, according to


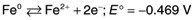
(1)

(revised redox potential; Appendix S1). In rusting, which to our present knowledge is a purely chemical (abiotic) process, oxygen accepts electrons (4e^−^ + O_2_ + 4H^+^ ⇄ 2H_2_O; *E*° = +1.229 V; *E*°′ = +0.815 V) and finally leads to the formation of brittle ferric oxides/hydroxides. Another ubiquitous electron acceptor is protons yielding hydrogen (2e^−^ + 2H^+^ ⇄ H_2_; *E*° = ± 0.000 V*; E*°′ = −0.414 V). However, this is technologically only serious in rare instances of acidic surroundings. Proton reduction in circumneutral H_2_O and thus the net reaction


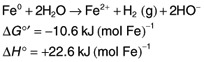
(2)

are very slow (Fig. S1) so that iron in sterile anoxic water can, in principle, last for centuries. The corrosion risk for iron in the absence of acid or oxygen changes dramatically if constructions are exposed to non-sterile, ‘environmental’ aqueous surroundings where microorganisms such as SRB can grow and obviously accelerate iron oxidation enormously (Hamilton, 2003). Iron loss rates of 0.2–0.4 mm Fe^0^ year^−1^ are typically recorded *in situ* (Jack, 2002; Table S1). Two basically different modes by which SRB act upon iron have been envisaged (Dinh *et al*., 2004).

First, undissociated protons in H_2_S from respiratory reduction of natural sulfate (e.g. in seawater) with organic nutrients react more rapidly with iron-derived electrons than do protons from or in (circumneutral) H_2_O, according to


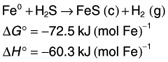
(3)

In such way, SRB act indirectly through an excreted chemical agent. We here refer to this process as ‘chemical microbially influenced corrosion’ (CMIC). The net reaction (Dinh *et al*., 2004) can be expressed, for instance with organic carbon of the oxidation state of carbohydrates (‘CH_2_O’, viz. the abundant building structure 〈H-C-OH〉) as


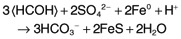
(4)

(details in Appendix S1).

Second, SRB can be involved more intimately in anaerobic iron corrosion by a mechanism that is fundamentally different from the above CMIC. This was first envisaged in a groundbreaking study of iron pipe corrosion in anoxic soil (von Wolzogen Kühr and van der Vlugt, 1934). SRB were suggested to use iron as the only source of reducing equivalents for sulfate reduction. The net stoichiometry of this purely lithotrophic process, here with the common carbonate (siderite) precipitation, is


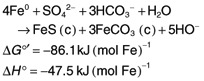
(5)

Still, such bulk equation cannot provide hints as to the actual form of the reducing equivalents channelled from iron into sulfate reduction. It has been appealing to consider H_2_ (from H_2_O reduction; Eq. 2) as the intermediate (Booth and Tiller, 1960; von Wolzogen Kühr, 1961; Bryant and Laishley, 1990; Coetser and Cloete, 2005), indeed an excellent growth substrate of many SRB. Their high-affinity hydrogen scavenging (4H_2_ + SO_4_^2−^ + 2H^+^ → H_2_S + 4H_2_O) is thought to ‘pull’ the primary oxidation (von Wolzogen Kühr and van der Vlugt, 1934; von Wolzogen Kühr, 1961), an explanation also common in textbooks. On the other hand, accelerated anaerobic corrosion due to H_2_ utilization has been viewed critically (Costello, 1974; Hardy, 1983). In several kinetic studies, H_2_ scavenging did not accelerate iron oxidation (Spruit and Wanklyn, 1951; Dinh *et al*., 2004; Mori *et al*., 2010). Furthermore, novel marine deltaproteobacterial SRB enriched and isolated directly with metallic iron as the only electron donor reduced sulfate much faster than possible by mere scavenge of H_2_ and were more corrosive than conventional strains (Dinh *et al*., 2004). Moreover, they transiently formed much H_2_ rather than scavenged it, possibly due to an initial excess of iron-derived reducing power. Therefore, the ability to make use of Fe^0^ for sulfate respiration in a kinetically more efficient manner than via the slowly formed abiotic H_2_, viz. through a faster by-pass, was assumed, and direct electron uptake from iron has been suggested (Dinh *et al*., 2004). This theory is here referred to as ‘electrical microbially influenced corrosion’ (EMIC).

In this study, we investigated the extent of iron destruction by these strains of SRB as well as the postulated EMIC and its significance in more detail. First, we measured whether corrosion rates as high as observed in industrial settings can be also attained *in vitro* by appropriately adjusted cultivation conditions. Second, we examined whether and in which way the increasing coverage of the metallic substrate by the inorganic black corrosion crust (Dinh *et al*., 2004) is compatible with progressive corrosion and the hypothesized electron uptake from the metal. Third, we buried iron specimens in a field study in natural marine sediment to prove whether corrosion phenomena *in situ* were similar as observed in laboratory incubation experiments.

## Results

To study the postulated EMIC by the previously isolated strains under experimentally defined conditions, metallic iron was provided in the form of coupons as the sole electron donor for sulfate reduction. The only added organic compounds were trace amounts of vitamins (totally 0.58 mg l^−1^, Table S2), and acetate (1 mM) provided as a biosynthetic building block to lithoheterotrophic strains IS5, HS3, and to *Desulfopila inferna*. Cultures incubated with 10 mM acetate without iron did not produce any sulfide, indicating that external acetate was not used as an electron donor. Measures of corrosion were the determination of iron mass loss at the end of incubation, a long-established routine method (Booth *et al*., 1967), and quantification of sulfate consumption, a more recently established method (Dinh *et al*., 2004) allowing highly resolved time-courses. Consumption of sulfate parallels production of sulfide that cannot be monitored directly due to precipitation as FeS (Eq. 5). An analytical control experiment verified that disappearance of sulfate was only due to reduction and not in addition to a certain co-precipitation in the forming corrosion crust (Fig. S2).

### Iron corrosion rates in long-term incubation experiments

**Fig. 1:**
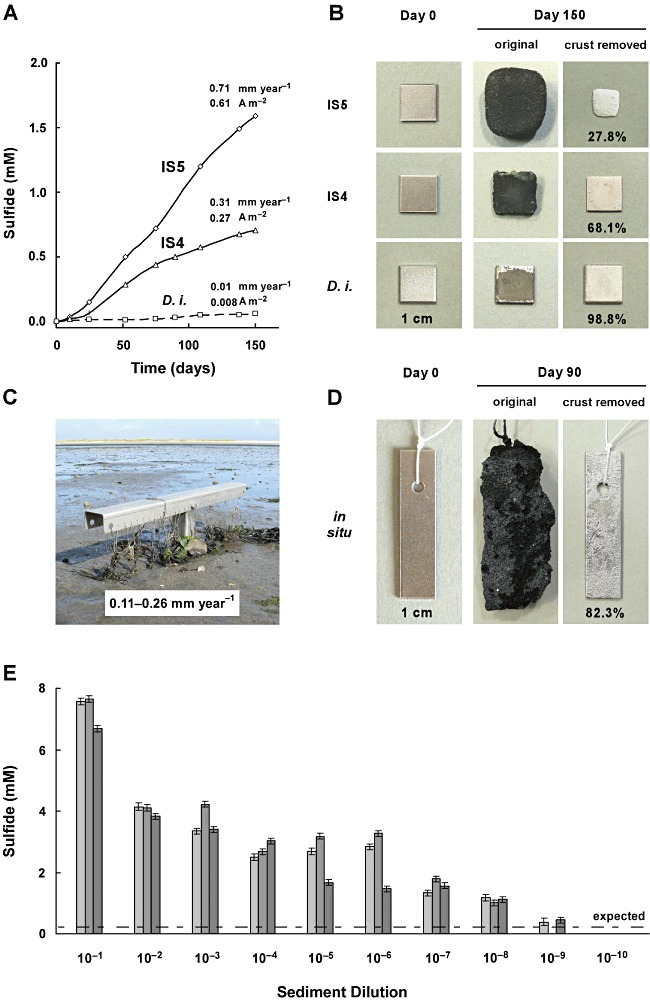
Corrosive sulfate-reducing bacteria in pure cultures and *in situ*. A. Long-term sulfide formation (measured as sulfate consumption) with iron coupons as the only electron donor in cultures of corrosive strains IS5 and IS4 (proposed: *Desulfovibrio ferrophilus* and *Desulfopila corrodens* respectively), and in a hydrogenotrophic control culture (*Desulfopila inferna*, *D*. *i*.). B. Thick corrosion crusts and metal loss in the same cultures. Residuary metal (% of initial) became obvious after crust removal by HCl-hexamine. C. Positioning device (stainless steel) for iron coupons in the Wadden Sea, island of Sylt (North Sea). Iron coupons were bound with threads to the device and buried for three months at ≥ 20 cm depth in anoxic sediment. D. Corrosion crust (with sand grains), and corroded metal (after crust dissolution) from (C). Here, the photographed fresh coupon (Day 0) is not the same as the incubated one. E. Sulfide formed (measured as sulfate consumed) after six months in serial dilutions (three in parallel) with native sediment (2 g, wet mass) from the same habitat. The line indicates sulfide expected solely by consumption of H_2_ formed from iron and seawater (based on independently measured H_2_-formation rates and experiments with merely H_2_-scavenging SRB).

Metallic iron represents a very compact, dense form of an electron donor sufficient to reduce dissolved sulfate from a relatively large culture volume. In the initial study (Dinh *et al*., 2004), the culture volume (0.15 l) to metal (30 g) ratio was kept relatively small for clearly revealing the corrosive potential of novel marine SRB within 20 days. In such incubations, the sulfate reduction rate slowed down significantly after a while. Examination in more detail in the present study revealed that this drop in activity was mostly due to the pronounced alkalization and exhaust of counteracting CO_2_ (dissolved and gaseous). For the present biocorrosion experiments intended to examine iron destruction under conditions comparable to those *in situ* during much longer incubation, the ratio of the culture (and gas phase) volume to metal mass had to be increased. Because macroscopic corrosion phenomena were of central interest, the iron specimens (10 mm × 10 mm × 1 mm) could not be miniaturized to any extent, thus necessitating much bigger culture volumes. An appropriate medium volume was 1.4 l, which was still small enough for precise monitoring of sulfate consumption. Indeed, corrosion rates did not significantly decrease over months. Corrosive cultures reached values as high as 0.7 mm Fe^0^ year^−1^ and deposited steadily growing black crusts (Fig. 1A and B). After selective crust removal, severe metal loss was evident (Fig. 1B). In the present experiments, strain IS5 was more corrosive than strain IS4, whereas in the initial physiological characterization (Dinh *et al*., 2004), the latter was more corrosive. This may be due to the higher tolerance of strain IS4 to the significantly increasing pH in the previous incubations. ‘Conventional’ SRB (control strains), which were *Desulfopila inferna* (a phylogenetic relative of strain IS4; Gittel *et al*., 2010), and *Desulfovibrio* strain HS3 (an effective scavenger of H_2_ isolated in this study), showed essentially no signs of iron corrosion within the incubation period. Iron in these control cultures was not more affected than in sterile incubations (Fig. 1A and B; Fig. S3). The inability to make more efficient use of iron was not due to sensitivity towards Fe^2+^ ions. The control strains were able to scavenge H_2_ formed from iron and water (Eq. 2) below detection limit (40 ppmv) and grew readily in the presence of iron if H_2_ was supplied externally (Fig. S4).

### Localization of corrosive cells, and determination of crust conductivity

If the pronounced corrosion is due to direct electron uptake by SRB, cells must be always electrically connected to their metallic substrate. This could be possible by direct attachment to the metal. However, such localization would implicate increasing coverage by the forming hard corrosion crust and cut-off from the medium which supplies sulfate and counteracts the strongly alkalizing effect of iron oxidation (Eq. 5). Progressive utilization of metallic iron despite coverage by crust would be possible if active cells would colonize the medium-exposed crust surface, and if the crust would be electrically conductive.

**Fig. 2:**
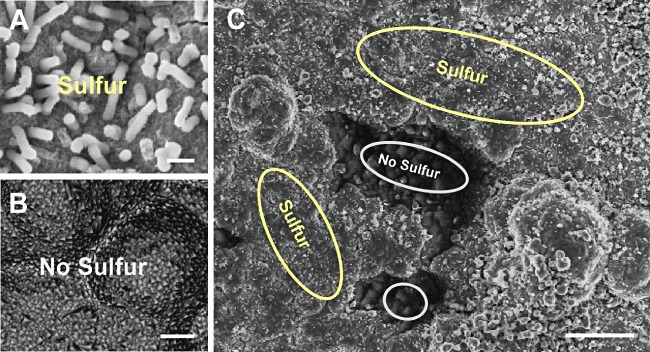
X-ray microanalysis (EDX) of crust surface in a culture of corrosive strain IS4. A. Site colonized by cells (Bar, 1 µm). B. Site without microbial colonization (Bar, 1 µm). C. Both sites in the same field of view (Bar, 20 µm). Surface-attached cells of strain IS4 colocalize with the element S. Cells were not detectable at sulfur-free sites. Both, sulfur-containing and sulfur-free sites contained the elements Fe, C and O. Sulfur-free sites contained in addition Mg and Ca. Thirty point spectra at 10 kV were collected for each site. Resolution (lateral and vertical), 3–5 µm.

Indeed, virtually no planktonic (free-living) cells could be observed, and scanning electron microscopy revealed a densely colonized crust surface in the corrosive cultures of strains IS4 or IS5. Colonized areas of the structurally heterogeneous crust contained the element S in addition to Fe, C and O (details in Fig. 2), as revealed by energy-dispersive X-ray spectroscopy (EDX) of the uppermost (*c*. 5 µm) crust. Sulfur-free patches were never colonized.

**Fig. 3:**
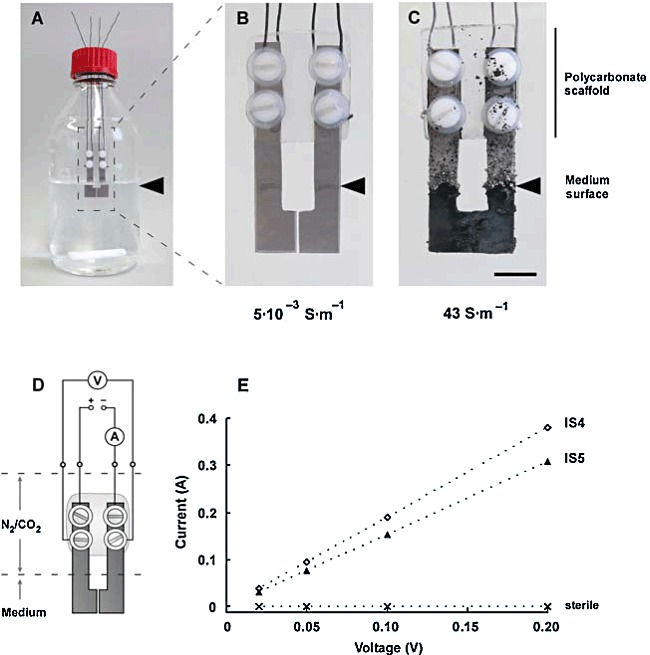
Determination of the conductivity of the corrosion crust. A. Anoxic bottle (600 ml medium) with two specially shaped fresh iron coupons. B. Coupons after three weeks of incubation in sterile medium. C. Coupons after three weeks of incubation with strain IS4. Bar, 1 cm. D. Scheme of the arrangement with voltage control and current measurement through separate circuits. E. Linear response of current to applied (non-electrolytic) voltage (0.02–0.2 V, DC).

Crust conductivity was evaluated as follows. Iron granules employed in previous cultivation (Dinh *et al*., 2004) tended to be cemented by the developing crust. This feature opened a simple way to measure conductivity of the crust in a non-invasive manner if the precipitate was allowed to cement two iron coupons fixed at defined distance and connected to monitoring wires protruding the stopper of the anoxic flask (Fig. 3, Fig. S5). The mounted coupons were only partly immersed so as to keep iron other than the slot-forming part outside of the medium. The conductivity of the biogenic crust measured at a voltage (< 0.2 V; DC) far below that for water electrolysis was around 50 S m^−1^ (Fig. 3, Table S3).

### Bulk composition and surface structures of biogenic corrosion crust

The bulk composition of the crust formed by strain IS4 was analysed quantitatively by combining EDX, X-ray diffraction (XRD), inductively coupled plasma optical emission spectroscopy (ICP-OES), and infrared spectroscopy. This revealed siderite (FeCO_3_) and amorphous ferrous sulfide at the expected ratio (Eq. 5; Table 1), and additional co-precipitated minerals such as calcite (CaCO_3_).

**Table 1:** Analysis of corrosion products of iron coupons from cultures of strain IS4, and from burial in permanently anoxic sulfidic marine sediment (Sylt, North Sea)

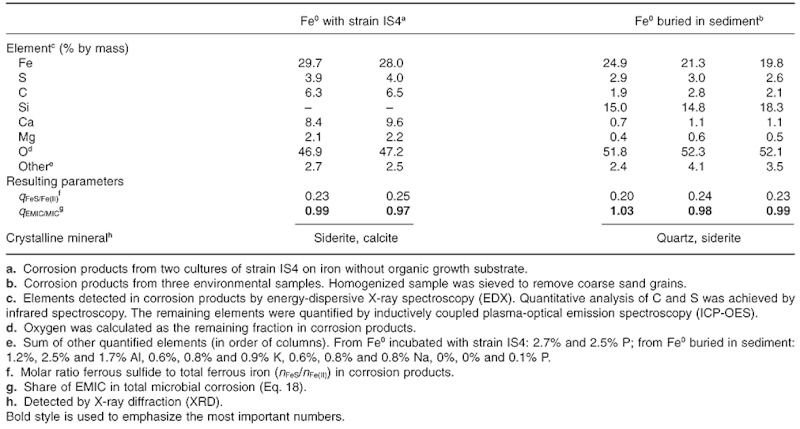

**Fig. 4:**
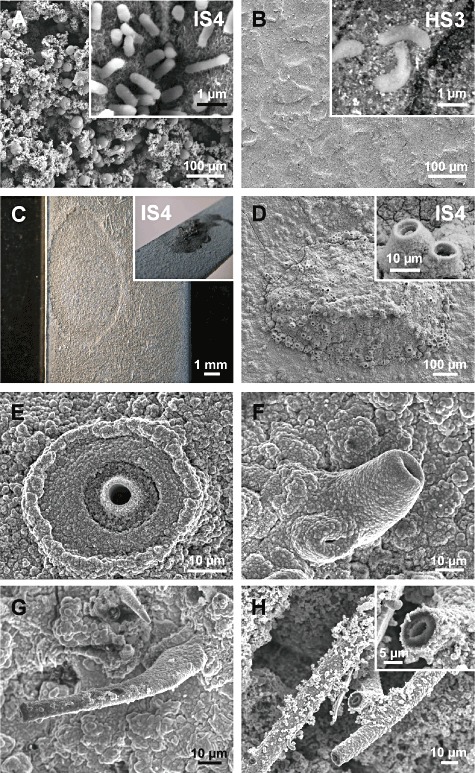
Scanning electron micrographs of crust surface, colonization and microchimneys. A. Cells of strain IS4 on corrosion crust after three months. B. Cells of non-corrosive hydrogenotrophic control strain HS3 on slightly corroded metal surface after three months. C. Anodic iron dissolution (crust removed) underneath a ‘pustule’ (insert) formed by strain IS4 at high pH (≍ 9; Fig. 5C). D. ‘Pustule’ with short microchimneys (magnified in insert) in a culture of strain IS4. E. Microchimney developing from a crater-like structure in a culture of strain IS4. F. Short microchimney in a culture of strain IS4. G. Long microchimney in a culture of strain IS4. H. Long microchimney (magnified in insert) in an alkalizing corrosive enrichment.

Figure 4 shows various images of the corrosion crust or coupon surface. On the crust covering coupons in cultures with strongly increased pH (as often observed in small culture volumes), ‘pustule’-like elevations appeared after several weeks of incubation (Fig. 4C, insert). The iron located underneath such ‘pustules’ exhibited a pronounced pitting area, as visualized upon crust removal (Fig. 4C). Strikingly shaped microscopic structures emerged on top of such ‘pustules’ at pH ≥ 9. In such cultures, the otherwise irregular crust exhibited round crater- or chimney-like structures (Fig. 4D–H, Fig. S6–8). Various growth stages of these structures were observed (Fig. 4).

### Field study of iron corrosion in marine sediment

To examine as to which extent the metallic iron used in laboratory experiments undergoes corrosion in a natural environment with sulfate reduction, iron coupons were buried in the dark (anoxic) part of a silty marine mud flat (Wadden Sea, island of Sylt, Germany) at *c*. 20 cm below the surface. Recovery was ensured by fixation via threads to a T-shaped positioning device (Fig. 1C). The coupons retrieved after three months were covered by thick black crusts (Fig. 1D). Their enormous thickness was largely due to sedimentary minerals (e.g. sand) cemented with the corrosion crust. Again, a characteristic crust composition of siderite and amorphous iron sulfide (Table 1) as well as surface pitting and mass or thickness loss ofthe metal were evident (up to 0.26 mm Fe^0^ year^−1^; Fig. 1D).

In addition, the natural abundance of SRB with corrosive potential was estimated via dilution series in anoxic tubes with iron coupons as the sole electron donor. The examined sediment sample was taken from the same site before the coupons were buried, viz. there was no artificial pre-enrichment of SRB with metallic iron. Development of sulfate reduction to a higher extent than would be possible by mere scavenging of chemically formed H_2_ (known from sterile control incubations) indicated corrosive SRB at numbers of more than 10^7^ cells per gram wet sediment (Fig. 1E).

## Discussion

In the present study, the ability of SRB to utilize metallic iron lithotrophically and thus cause corrosion was even more pronounced than previously expected (Fig. 1; Dinh *et al*., 2004). Apparently, only particular species of SRB can effectively exploit iron as an electron donor for fuelling their energy metabolism through sulfate reduction, so that distinction between corrosive and non-corrosive (‘conventional’) strains or species is justified. Corrosive SRB are not necessarily related on the basis of 16S rRNA-based phylogeny; they branch within distinct lineages of SRB (Dinh *et al*., 2004). Nevertheless, more extended comparative corrosion studies are needed to clarify whether corrosiveness is a genetically fixed trait, or whether also ‘conventional’ strains, for instance close relatives of strains IS4 and IS5, can gradually adapt to utilize and corrode iron if exposed to the metal over years. The ability to utilize iron directly as an electron donor for sulfate reduction primarily urges upon an understanding of the underlying mechanisms.

### Towards an understanding of the corrosion mechanisms

Principal physico-chemical considerations as well as previous (Dinh *et al*., 2004) and present incubation experiments together with the measured electrical conductivity of the corrosion crust are strongly in favour of the EMIC hypothesis, that is direct electron gain for sulfate respiration from the metal via the crust.

The reduction of H^+^-ions (strictly, H_3_O^+^-ions) by Fe^0^-derived electrons on the metal surface is a par excellence example of a kinetically ‘impeded’, slow electrochemical reaction. Availability of H^+^ ions at the metal surface and combination of the primarily formed atomic hydrogen [e^−^ + H^+^ → H_(adsorbed)_] to H_2_ are commonly understood as kinetic ‘bottle neck’ that also explains the high negative electrochemical overpotential (difference between potential during net reaction and equilibrium potential under the given conditions) of electrochemical H_2_-formation on iron (Bockris and Reddy, 1970; Hamann *et al*., 2007). Microbial scavenge of H_2_, viz. a product behind the ‘bottle neck’, is therefore not expected to accelerate the primary iron dissolution (Fig. S1). This is in accordance with experimental findings in evaluations of the ‘cathodic hydrogen’ theory of MIC (Costello, 1974; Hardy, 1983; Dinh *et al*., 2004). Some corrosive strains even formed significantly higher amounts of H_2_ than sterile incubations during the initial incubation phase with metallic iron. Deposition of some black FeS at the glass walls of the bottles indicated that a part of the SRB population grew distantly from the coupons with such biologically released H_2_. The assumption of a direct electron uptake by cells would not only explain the high corrosion rate of special SRB, but also the pronounced initial release of H_2_. This is possibly an ‘unavoidable’ side reaction via a hydrogenase because electron uptake from freshly supplied iron may be faster than electron consumption by sulfate reduction (Dinh *et al*., 2004).

Since electrons, unlike chemical compounds such as H_2_, cannot diffuse or flow through water, electron-conducting structures would be needed. On the side of the cell, these might be outer membrane and periplasmic membrane proteins investigated in various microorganisms in bioleaching of metals (Appia-Ayme *et al*., 1999; Auernik *et al*., 2008), extracellular iron(III) reduction or microbial fuel cells (Butler *et al*., 2010). Between cells and the corroding iron, which is being covered by a steadily growing sulfidic corrosion crust, the latter itself is envisaged as the electrical mediator. Metal sulfides, which tend to be non-stoichiometric, are long-known semiconductors (Braun, 1875; Pearce *et al*., 2006), and some earlier biocorrosion models based on H_2_ production and consumption hypothesized about a participation of semiconductive FeS (Booth *et al*., 1968; King and Miller, 1971) in abiotic H^+^ reduction.

The undisturbed corrosion crust in cultures of strains IS4 and IS5 indeed exhibited a conductivity of around 50 S m^−1^ (A V^−1^ m^−1^); this is even higher than that of many typical semiconductors (e.g. pure silicon, 1.6 × 10^−3^ S m^−1^; Table S4) or microbial biofilms with nanowires allowed to form between gold sheets mounted in cultures of *Geobacter sulfurreducens* (0.5 S m^−1^; Malvankar *et al*., 2011). Conductivity of the heterogeneous corrosion crust must be due to contained iron sulfides because FeCO_3_ and CaCO_3_ are essentially insulating minerals (10^−10^ and 10^−14^ S m^−1^ respectively; Table S4). This was confirmed in the present study by a conductivity test of siderite mineral (Fig. S9). Even though the measured biocorrosion rates of 0.71 mm Fe^0^ year^−1^ were high and technologically relevant, the corresponding current density of 0.61 A m^−2^ (Appendix S1) would need a voltage (potential difference) of only *V* = 1.2 × 10^−4^ V across a 1 cm crust. The calculated equilibrium potential at the corroding iron surface and the zone of sulfate reduction is around −0.60 and −0.25 V, respectively, viz. Δ*E* = 0.35 V (couples FeCO_3_/Fe^0^ and SO_4_^2−^/FeS respectively; Appendix S1). Hence, there is significant leeway for the ‘self-adjusting’ potential difference driving the corrosion current through the crust. Crust conductivity is apparently not a rate-limiting factor. The model of corrosive SRB gaining electrons through semiconductive ferrous sulfide is further corroborated by the electron microscopic finding of cells attached mostly to the sulfide-rich islands within the predominantly carbonaceous structure (Fig. 2).

**Fig. 5:**
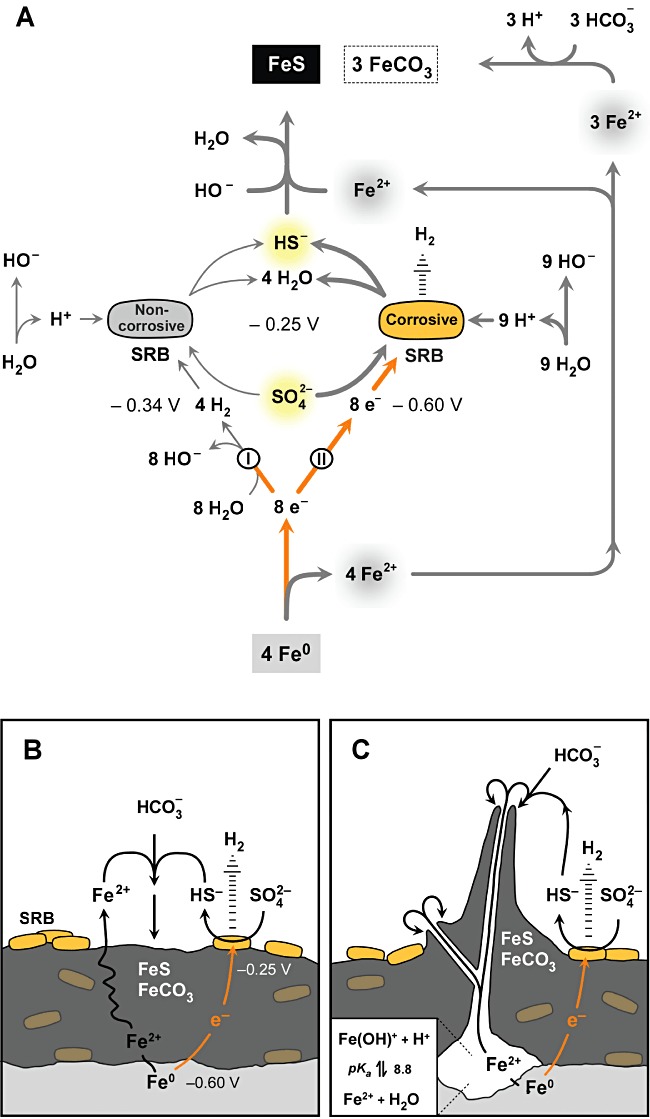
Scheme of the stoichiometry and topology of direct (lithotrophic) corrosion. A. Stoichiometry of iron dissolution and channelling of electrons via H_2_ (I; classical scheme) or directly (II; new model) into sulfate reduction. Bold lines indicate the much faster electric ‘bypass’. Equilibrium redox potentials are indicated for real conditions at *pH* = 8 (see Appendix S1). Direct electron utilization provides a higher metabolic driving force (voltage: Δ*E* = 0.35 V, compared with 0.09 V via H_2_ of assumed ≍ 40 ppmv). Fe^2+^ not precipitated as sulfide may enter solution or precipitate with naturally widespread inorganic carbon as FeCO_3_. B. Electron flow through the crust to attached cells at *pH* = 8 (simplified, non-stoichiometric). Crust may contain co-precipitated calcium and magnesium carbonate, and/or cemented sand. The equivalent ion flow may occur via aqueous interstices (not depicted). C. Build-up of chimney-like ion bridges at *pH* ≥ 9. Reactions are essentially as in (B); however, there are pronounced spots of anodic iron dissolution. A significant *pH* gradient (low inside, high outside) causes Fe(II) precipitation at the rim, leading to chimney growth. Schemes include the possibility of H_2_ release (that may foster remote bacterial cells) due to an imbalance between electron uptake and sulfate reduction. Participation of possibly buried (encrusted) cells in sulfate reduction and H_2_ release is unknown.

Electrons can only flow to cells if the crust also allows an equivalent ion flow via aqueous ‘bridges’ (maintenance of electroneutrality). These may be tiny interstices or fissures. An apparent, striking ion bridge, also strongly supporting the model of transcrustal electron flow, emerged at *pH* ≥ 9. In such cultures, the otherwise irregular crust exhibited round crater- or chimney-like structures (Fig. 4D–H, Fig. S6–8). Their formation is presently explained by a lowered, crust-preventing *pH* inside due to slightly acidic Fe^2+^-ions [Fe^2+^ + H_2_O ⇄ Fe(OH)^+^ + H^+^; *pK_a_* = 8.8] and precipitation of Fe^2+^ as soon as it enters the high-*pH* carbonate-containing medium (Fig. 5C, Fig. S10).

In conclusion, anaerobic corrosion caused by the direct, lithotrophic mode of iron utilization according to Eq. 5 can be only explained by direct electron uptake (Fig. 5), i.e. real occurrence of the electrochemical half-reaction


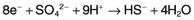
(6)

(*E*°′_av_ = −0.218 V, average) coupled to iron dissolution (Eq. 1). Hence, the lithotrophic, direct corrosion is always EMIC.

### Comment on direct corrosion by methanogenesis

There is first evidence that also special methanogenic archaea obtained through enrichment with metallic iron as the only source of reducing equivalents can bypass the slow abiotic H_2_ formation on iron in water by faster direct use of the electrons (Eq. 1) according to 8e^−^ + HCO_3_^−^ + 9H^+^ → CH_4_ + 3H_2_O (Dinh *et al*., 2004; Mori *et al*., 2010; Uchiyama *et al*., 2010). Here, the net reaction, 4Fe^0^ + 5HCO_3_^−^ + 2H_2_O → 4FeCO_3_ (c) + CH_4_ (g) + 5HO^−^[Δ*G*°′ = −73.9 kJ (mol Fe)^−1^; Δ*H*° = −35.3 kJ (mol Fe)^−1^], does not lead to a conductive precipitate. One may speculate that in this case cell-metal contact must be sustained so that hindrance by crust coverage may become obvious during long-term incubations (which have not been carried out so far). Nevertheless, the process may play a role in MIC because methanogenic archaea may take advantage of electroconductive FeS precipitated by co-occurring sulfate reduction. In axenic laboratory cultures, there is some FeS precipitation by sulfide added as reductant.

### Biosynthesis during direct iron corrosion by sulfate reduction

An understanding of the properties of the precipitate formed during corrosion also requires knowledge of the proportion of formed cell mass that may be embedded. Because there is presently no convenient method for determining cell mass in the solid corrosion crust, its organic content was estimated. According to the principle of bifurcate substrate flow in every chemotrophic organism, the amount (e.g. in mol or mmol) of total iron oxidized in cultures of SRB performing EMIC, *n*_FeEMIC_, is the sum of the amount oxidized catabolically by sulfate reduction, *n*_FeCatab_, and the amount oxidized due to the anabolic need of electrons, *n*_FeAnab_, i.e.


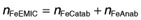
(7)

(basic scheme in Fig. S11). If EMIC is the only corrosion process, *n*_FeEMIC_ is identical with the loss of metallic iron, *n*_ΔFe(0)_, and *n*_FeCatab_ is fourfold higher than the amount of sulfate being reduced (Eq. 5), viz. *n*_FeCatab_ = 4 *n*_SR_. Hence, anabolic iron oxidation can be expressed as the difference of two measurable parameters according to


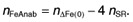
(8)

With this, also a partition coefficient (quotient) or contribution of anabolic iron oxidation to total iron oxidation during EMIC can be formulated as


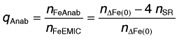
(9)

Iron loss is equivalent with ferrous iron formation, i.e. *n*_ΔFe(0)_ = *n*_Fe(II)_. The resulting *n*_FeAnab_ (Eq. 8) can betranslated into formed biomass via assimilation equations if an elementary bulk composition and hence a formula mass (‘molecular’ mass) of bacterial dry mass is assumed. The assimilation equations express how much cell mass, *m*_Bio_ (e.g. in g or mg), is formed per amount of iron used for the anabolism, viz. they allow to formulate an anabolic yield coefficient,


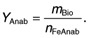
(10)

Here we used the simplified bulk formula C_4_H_8_O_2_N (van Dijken and Harder, 1975; comments in Widdel and Musat, 2010) with *‘M’* = 102.1 g mol^−1^. Synthesis of cell carbon may occur with CO_2_ alone (lithoautotrophic growth; strain IS4), or require in addition an organic substrate such as acetate (lithoheterotrophic growth; strain IS5). The resulting assimilation equations are


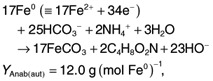
(11)

and


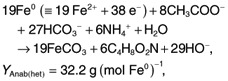
(12)

for autotrophic and heterotrophic growth respectively. The latter equation is based on the observation that *c*. ⅔ of cell carbon in SRB is derived from acetate and *c*. ⅓ from bicarbonate (Sorokin, 1966; Badziong and Thauer, 1978). Through such theoretical assimilation equations and Eq. 8, the expected biomass can be calculated from *n*_FeAnab_ as


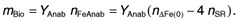
(13)

The mass of the minerals (FeS, FeCO_3_, co-precipitated CaCO_3_ and possibly MgCO_3_) precipitated during lithotrophic corrosion, *m*_Min_, can be calculated from the same measurable parameters as *n*_FeAnab_ (Eq. 8), viz. from *n*_ΔFe(0)_[or *n*_Fe(II)_] and *n*_SR_ (Appendix S1). This further allows to express the biomass content as a partition coefficient (quotient) relating the biomass, *m*_Bio_, to the total mass of precipitated crust, *m*_Min_ + *m*_Bio_. For this, the formulas


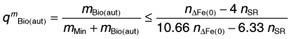
(14)

and


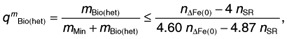
(15)

can be derived (Appendix S1) for autotrophic and heterotrophic growth respectively (again with *n*_ΔFe(0)_ = *n*_Fe(II)_). They are applicable if EMIC is the only process of corrosion, as in our cultures, and if one assumes complete precipitation of ferrous iron (and variable precipitation of Ca/MgCO_3_).

Strains IS4 and IS5 corroded *n*_▵Fe(0)_ = 4.31 and 9.86 mmol Fe^0^, respectively, during reduction of *n*_SR_ = 0.95 (± 0.06) and 2.14 (± 0.08) mmol SO_4_^2−^ respectively. Hence, the amounts accounting for biosynthesis (Eq. 8) of strains IS4 and IS5 were *n*_FeAnab_ = 0.51 and 1.3 mmol Fe^0^, respectively, which yields the following values for partition of anabolic in total iron oxidation (Eq. 9), and for the biomass content of the crust (Eqs 14 and 15):


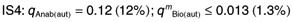
(16)


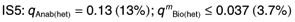
(17)

These results indicate that the bulk of electrons is channelled into the catabolism (sulfate reduction), as common in the strictly anaerobic SRB, and that the corrosion crust is essentially inorganic, in agreement with its hard, mineral-like appearance. The corrosion crust thus profoundly differs from typical biofilms that are largely constituted of cells and an organic matrix.

### Practical considerations

Protection of iron against corrosion by alloying or coating is technically and economically not possible at any extent. For instance, the most important alloying metal, chromium, is produced at a 48-fold lesser extent than iron (US Geological Survey, 2011; data for 2009). We therefore expect that advances in the analysis and control of MIC *in situ* will remain a relevant issue.

A long-term goal would be the design of specific counter measures based on detailed understanding of the environmental conditions and mechanisms leading to MIC. Such a ‘causative’ approach is confronted with a complexity of chemical conditions (e.g. availability of electron acceptors, or electron donors in addition to metallic iron) and chemical, electrochemical and microbial reactions in MIC. The most promising research strategy is, in the opinion of the authors, an experimental ‘dissection’ into individual and elementary processes for later synopsis. In the previous (Dinh *et al*., 2004) and present study, we focused on lithotrophic growth with metallic iron as the only electron donor under strictly anoxic conditions in the presence of sulfate as electron acceptor. Further, more comprehensive microbiological investigations would have to consider contributions to corrosion for instance by lithotrophic methanogenic archaea (Dinh *et al*., 2004; Mori *et al*., 2010; Uchiyama *et al*., 2010) if sulfate is limiting, or by CMIC due to organotrophic growth of SRB in organic-rich environments. Among the involved inorganic compounds, iron sulfides with their delicate, variable chemical and electrochemical properties are of central interest. They cannot only mediate electron flow. Under certain, high-sulfide conditions iron sulfides apparently protect the underlying metal against further rapid corrosion (Smith and Miller, 1975; Lee *et al*., 1995). Another, different role has been attributed to iron sulfides under intermittent anoxic-oxic conditions where corrosion can be particularly severe. This has been explained mainly by the reactivity of chemical oxidation products of ferrous sulfide towards iron (Jack, 2002; Hamilton, 2003; Beech and Sunner, 2007).

In view of the presently limited understanding of MIC *in situ*, recommendation of control measures is also limited. As long as MIC in a particular situation is largely CMIC, prevention would be possible if the organic nutrient of the anaerobic bacteria can be identified and eliminated. If MIC is essentially EMIC, avoidance of sulfate-containing water would be a causative solution. Extended *in vitro* and pilot studies may clarify as to which extent an addition of nitrate, which is applied to control biogenic H_2_S (souring) in petroleum reservoirs (Reinsel *et al*., 1996; Gieg *et al*., 2011), is also promising for controlling CMIC and EMIC (Hubert *et al*., 2005; Schwermer *et al*., 2008).

Despite the complexity of anaerobic corrosion processes *in situ*, the presently examined strains IS4 and IS5 are envisaged as representatives of the key players in MIC. These strains caused corrosion rates well matching those reported for the destruction of industrial iron structures in permanently anoxic aqueous surroundings (Table S1). Also, the rates of direct corrosion by strains IS4 and IS5 are similar as rates of chemical iron destruction by sulfide (Lee *et al*., 1995; Jack, 2002; Beech and Sunner, 2007; Table S1). This raises interest in an estimate of the contribution of EMIC in total MIC (EMIC plus CMIC by organotrophically formed sulfide) in cases of corrosion *in situ*. Because CMIC (Eq. 4) leads exclusively to sulfidic iron, whereas EMIC leads in addition to non-sulfidic (usually carbonaceous) iron, their measurable ratio in the crust of a corroded construction may be used for estimating the extent of EMIC. Such extent is quantitatively expressed as the quotient of the amounts (mol) of iron lost by EMIC, *n*_FeEMIC_, and iron lost totally by MIC, *n*_FeMIC_, and thus defined as *q*_EMIC_ = *n*_FeEMIC_/*n*_FeMIC_. Assuming that all ferrous iron in the crust results from metal corrosion, *n*_FeMIC_ = *n*_Fe(II)_, so that *q*_EMIC_ = *n*_FeEMIC_/*n*_Fe(II)_. Further calculation so as to substitute the unknown *n*_FeEMIC_ and introduce the measurable amount of precipitated sulfide, *n*_FeS_, leads to the formula (calculation in Appendix S1)


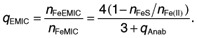
(18)

Hence, besides analysis of sulfidic and total ferrous iron in the crust, only the assumption of a *q*_Anab_ value (see above) is needed. For approximate calculation, *q*_Anab_ may be omitted (because *q*_Anab_ << 3). Still, such formal treatment is only applicable for anoxic conditions and absence of processes other than sulfate reduction, for instance methanogenesis (Dinh *et al*., 2004; Mori *et al*., 2010; Uchiyama *et al*., 2010), and absence of secondary conversion of FeCO_3_ to FeS (further remarks in Appendix S1). Analysis of the crust on the coupons recovered from the field study revealed *n*_FeS_/*n*_Fe(II)_ = 0.20–0.24. By assuming, for convenience, *q*_Anab_ ≍ 0.1, we obtain *q*_EMIC_ = 0.98–1.03 (theoretically, according to Eq. 18, *q*_EMIC_ always ≤ 1). This suggests that corrosion under the conditions prevailing at the studied marine sediment site was indeed only EMIC, viz. due to SRB capable of direct electron uptake. Such crust analysis (von Wolzogen Kühr and van der Vlugt, 1934; Spruit and Wanklyn, 1951), with awareness of its limits in view of additional processes, may be a more promising approach for understanding particular cases of corrosion than, for instance, the traditional analysis of aqueous phases (e.g. produced waters in oil fields). Because EDX as a semi-quantitative technique (Goldstein *et al*., 2003) is not applicable for determining mineral ratios in the crust, chemical analysis is the technique of choice.

The presence of SRB with the capability for EMIC such as strains IS5 and IS4 may be examined by a complementary cultivation-based approach. Such SRB may have been overlooked in microbiological monitoring studies of MIC which employ diagnostic methods based on fast growth with organic nutrients such as lactate and samples from water phases. The presently investigated corrosive strains grow relatively slowly and show pronounced surface attachment, so that they are easily out-competed in organic-rich diagnostic media by naturally widespread, rapidly growing planktonic SRB. Hence, development of convenient media with Fe^0^, inocula scraped off from surfaces, and longer than conventional incubation times should be envisaged.

If molecular, nucleic acid-based analyses of damages by microbial corrosion are of interest, again focus on the microorganisms in precipitated products rather than in aqueous phases is recommended for future studies (Skovhus *et al*., 2011). Still, molecular detection of directly corrosive SRB by long-established markers such as 16S rRNA or its genes is intrinsically limited. Already the first investigations into direct corrosion showed phylogenetic unrelatedness of corrosive SRB (Dinh *et al*., 2004). There is presently no target gene which among the more general marker genes of SRB (Stahl *et al*., 2007) could indicate the unique physiological capability for EMIC.

### Physiological and ecological significance of the ability for iron corrosion

**Fig. 6:**
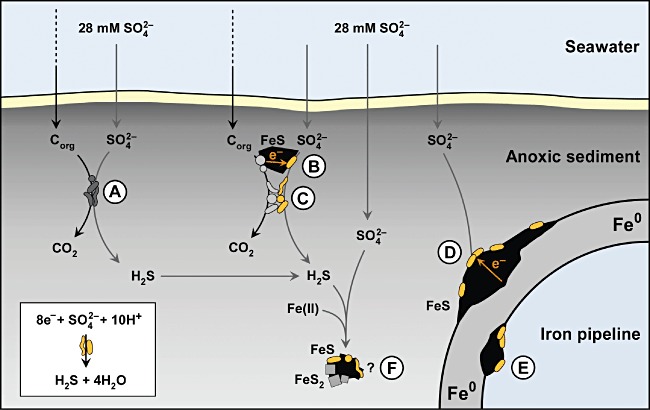
Present synoptic hypotheses of the role of sulfate-reducing bacteria (SRB) capable of electron uptake from external sources in anoxic marine sediment (not to scale). A. Conventional organotrophic SRB. B. SRB interacting via electroconductive ferrous sulfide with electron-donating organotrophic anaerobic microorganisms. C. SRB interacting in direct contact with electron-donating organotrophic anaerobic microorganisms. D. Special SRB exploit metallic iron as electron source at the outer surface of a pipeline. E. Special SRB exploit metallic iron as electron source inside of a pipeline. F. Speculative possibility of pyritization of FeS (FeS + H_2_S → FeS_2_ + 2H^+^ + 2e^−^) as a direct electron source for sulfate reduction.

The specific ability to utilize metallic iron as an electron donor is a physiologically striking capability, the ecological significance of which is presently unknown. Apart from rare cases (meteorites, seldom rocks from deep subsurfaces; Deutsch *et al*., 1977; Haggerty and Toft, 1985), metallic iron has been introduced into the environment on a large scale only by industrialization, viz. very ‘recently’ from an evolutionary point of view. Yet, counting of corrosive SRB via dilution series with anoxic sediment and metallic iron revealed several 10^7^ cells per gram wet mass (Fig. 1E), despite obvious absence of man-made iron constructions. One may speculate that corrosiveness represents the promiscuous use of a long-existing physiological trait for environmental electron uptake (‘electrotrophy’; Lovley, 2011) that is suited to also exploit the anthropogenically introduced metal as substrate. Normally, SRB with such trait may be involved in biogenic electron flow through sulfidic marine sediments and other ecosystems (Nakamura *et al*., 2009; Nielsen *et al*., 2010; Kato *et al*., 2011). Also, electron gain in direct contact with other bacteria with a surplus of catabolic electrons (Summers *et al*., 2010; Lovley, 2011) or from strongly reducing, reactive mineral surfaces, such as pyrite being formed from ferrous sulfide and free sulfide (FeS + H_2_S → FeS_2_ + H_2_ / FeS_2_ + 2H^+^ + 2e^−^; Wächtershäuser, 1992), can be envisaged as the genuine role of the electron uptake system underlying direct corrosion. Figure 6 summarizes a present hypothesis of the *in situ* function of SRB with the ability for electron uptake from external sources. Such SRB may represent a so far overlooked part of the anaerobic population. Still, more extended examinations (such as the above dilution series) of their abundance at various natural sites and physiological studies are needed to unravel their real significance in anaerobic mineralization.

From the viewpoint of mechanistic evolution, merely electron-donating and electron-accepting reactions can be regarded as simple or even primeval. Mere electron transfer does not require extra catalytic mechanisms like cleavage of C–H bonds (or of the H-H bond) and rearrangement of bound atoms. With many simple electron donors and acceptors, electron transfer takes place without specific catalysis simply according to redox potentials. This is a classical principle in biochemistry, for instance if electron accepting or donating dyes such as viologens or hexacyano-ferrates are used to react unspecifically with various redox proteins. In the cell, cofactors that can transfer electrons as such would be even critical in their free, dissolved form because of unspecific redox reactions. Their reducing or oxidizing power must be controlled for instance by embedding in a protein (haem, flavins) or by restriction to the lipophilic cytoplasmic membrane (quinones). Substances that merely accept or donate electrons are much less critical outside of the cell, and the ability to use them via transmembrane electron transport components is obviously a typical domain of environmental prokaryotes.

In conclusion, the study of microbial corrosion encompasses interesting mechanistic, ecological and evolutionary aspects, besides its obvious practical significance. It may become, besides microbial fuel cells (Lovley, 2006; Nealson and Finkel, 2011), microbial electrolysis cells for H_2_ production from H_2_O (Croese *et al*., 2011), and biogenic currents in environments (Nakamura *et al*., 2009; Nielsen *et al*., 2010; Kato *et al*., 2011), a fourth topic in the developing field of ‘electro-microbiology’ and in this way contribute to future synoptic views.

## Experimental procedures

### Organisms and cultivation

Strains IS4 and IS5, tentatively termed *Desulfopila*‘*corrodens*’ and *Desulfovibrio ‘ferrophilus*’, respectively, were re-activated from freeze-dried former cultures (Dinh *et al*., 2004). *Desulfopila inferna* was provided by Antje Gittel (University of Bergen, Norway; Gittel *et al*., 2010). SRB with high affinity for H_2_ were enriched from marine sediment with an H_2_-CO_2_ mixture (9/1, by volume) provided at growth-limiting rate through a silicon rubber membrane inside an anoxic cultivation device; some acetate (1 mM) was provided for cell synthesis. After two subcultures, the *Desulfovibrio* strain HS3 was isolated via agar dilution (Widdel and Bak, 1992).

Cultures were grown in CO_2_/bicarbonate-buffered artificial seawater medium (Widdel and Bak, 1992) in butyl rubber-stoppered bottles (routinely 150 ml) under an anoxic N_2_-CO_2_ (9/1, by volume) headspace at 28°C. SO_4_^2−^ was usuallyprovided at 28 mM; exceptions were cultures with 5 mM SO_4_^2−^ (not resulting in a noticeable decrease of the growth rate) for precise determination of its consumption. The reductant was usually Fe^0^ alone. However, some big cultures (1400 ml) received also 75 µM Na_2_S, which shortened the lag phase. All strains except strain IS4 were supplemented with 1 mM acetate for heterotrophic cell synthesis. H_2_-grown inocula were flushed with N_2_-CO_2_ to prevent transfer of H_2_ substrate and sulfide to Fe^0^ cultures. Iron specimens (mild steel EN 1.0330; > 99.37% Fe) were degreased with acetone (2 min), freed from oxide layers with 2 M HCl (2 min) and washed in sterile anoxic water (2 × 20 s). Coupons were then rapidly dried under N_2_, weighed (in sterile Petri dishes) and added to cultures. The ratio of medium volume to metal surface was between 15 ml cm^−2^ (for scanning electron microscopy; coupons: 30 mm × 10 mm × 1 mm) and 600 ml cm^−2^ (for corrosion rate determination).

Strain purity was routinely checked microscopically upon growth with H_2_ or lactate (+ yeast extract), and sequencing of 16S rRNA genes.

Cells of corrosive SRB in sediment were quantified via triplicate serial 1:10 dilutions with Fe^0^ as electron donor and 1 mM acetate as a carbon source. SO_4_^2−^ consumption was quantified after 6 months of incubation at 20°C. Sterile controls were included to calculate sulfate reduction solely based on the measured abiotic H_2_ formation. Marginal background sulfate reduction was measured in iron-free controls.

### Processing of corroded iron coupons

Corrosion crust for analysis was (inside an N_2_ chamber) scraped off the water-rinsed and dried coupons, finely ground in a mortar, and kept under anoxic N_2_ until analysis to prevent secondary oxidation. Corrosion coupons for weight loss determination were freed from the crust in aqueous 2 M HCl and 0.7 M hexamethylenetetramine (hexamine). The freed iron was washed in anoxic water and dried under N_2_ before weighing.

### Determination of corrosion crust conductivity

Shaped and polished mild steel coupons were fixed at exactly defined distance on a polycarbonate support (Fig. 2A and B). The four wires protruding the stopper of the anoxic flask (1 l, 0.6 l medium) for current and voltage control were also made of iron. Coupon-wire contacts were fixed with plastic screws. The device was sterilized with ethanol (2 h; dried afterwards). The lower, defined slot part was subjected to the pre-treatment of coupons described above and then immersed in artificial seawater medium (Fig. 2A and B). The medium was gently stirred during incubation. For conductivity measurement before and after crust formation, the current responding to 20, 50, 100 and 200 mV was measured. The instruments connected for this purpose were a TS3022S precision current (DC) supply (Thandar Instruments), an ammeter (± 0.2%, ± 10 µA), and a voltmeter (± 0.05%, ± 10 µV). Electrical conductivity was calculated as *σ* = *I d/*(*V a*); *I*, current; *d*, distance between coupons; *V*, voltage; *a*, split area (split height × coupon thickness). For photographic documentation, the mounted coupons were transferred to anoxic water.

The specific conductivity of siderite mineral (> 85% crystalline siderite with accompanying calcium and magnesium carbonates) was determined with specimens compressed (9 × 10^8^ Pa) to smooth pills in a type 15.011 hydraulic press (Specac Ltd). Impedance was measured via a ‘piston electrode’ (Fig. S9) connected to a Model 1286 potentiostat with a 1255B frequency response analyser and a 1281 multiplexer (Solartron Analytical).

### Field experiment

Mild steel coupons fixed by threads to a T-shaped scaffold (stainless steel; Fig. 1C) were buried during summer 2010 at 20 cm depth in silty black, anoxic sediment of the Wadden Sea (Tonnenlegerbucht, island of Sylt, Germany). Coupons recovered after three months were immediately transferred to anoxic artificial seawater and further processed as described for other coupons.

### Scanning electron microscopy

Crust-covered iron specimens were fixed with 2% (v/v) glutaraldehyde in anoxic artificial seawater at 4°C (12 h), washed with anoxic seawater, dehydrated with anoxic ethanol at increasing concentration (25%, 50%, 75%, 90%, 100% and again 100%, v/v; each step 2 min), pre-dried chemically with hexamethyldisilazane (30 min; Araujo *et al*., 2003), and fully dried under N_2_. Scanning electron microscopy (SEM) at 5–15 kV was performed with a Leo 1550 FE instrument (Zeiss).

### Mineral and solute analyses

For element identification, finely ground crust homogenate (see above) was compressed (9 × 10^8^ Pa) to smooth pills in a type 15.011 hydraulic press (Specac Ltd) and surface-mapped (using triplicate samples) by energy-dispersive X-ray microanalysis (EDX) at 10 kV in combination with SEM. Data were analysed with the INCA software (Oxford Instruments). C and S were subsequently quantified after combustion (to CO_2_ and SO_2_ respectively) using a CS-444 infrared spectrometer (Leco). Na, Mg, Al, Si, P, K, Ca and Fe were quantified by inductively coupled plasma optical emission spectroscopy (ICP-OES) with an IRIS Intrepid HR Duo instrument (Thermo Fisher Scientific). Si was quantified after alkaline pulping. The O-content was calculated as the remaining fraction. H (not detectable with the used instruments) is assumed to constitute a marginal fraction of the dried mineral crust.

Crystalline mineral phases in finely ground corrosion crust were identified by means of X-ray diffraction (XRD) analysis in Bragg-Brentano geometry with CuK_α_ radiation from 1 µm aperture in a D8 Advance diffractometer (Bruker). The 2*θ* angle was increased in 0.08° steps with 15 s counting time from 20 to 74.08°; the total scan time per diffractogram was 169 min. Data were analysed with the EVA software (Bruker).

H_2_ was quantified in samples withdrawn (with syringes through hypodermic needles) from the culture headspace via a GC-8A gas chromatograph (Shimadzu) equipped with a Porapak Q N80/100 (Machery-Nagel) column (temperature, 40°C; carrier gas, N_2_) and a thermal conductivity detector.

SO_4_^2−^ in filtered (0.45 µm pores) FeS-free samples was quantified via a 761 Compact Ion Chromatograph (Metrohm) by conductivity detection. Ions were separated via a Metrosep A Supp 5–100 column with an eluent of 3.2 mM Na_2_CO_3_ and 1 mM NaHCO_3_ at 0.7 ml min^−1^. Fe^2+^ dissolved in such filtrates was quantified by ICP-OES (see above).

## List of symbols and some abbreviations

**Table:**

CMIC	Chemical microbially influenced corrosion (indirect corrosion)
Δ*G*°	Change of free energy at 298.15 K and standard activities (numerically ≍ 1 M, 1 atm)
Δ*G*°′	As Δ*G*°, except {H^+^} = {HO^−^} = 10^−7^ (*pH* = 7)
Δ*H*°	Change of enthalpy at 298.15 K
EMIC	Electrical microbially influenced corrosion (direct corrosion)
*E*	Redox potential
*E*°	Redox potential at 298.15 K and standard activities (≍ 1 M, 1 atm)
*E*°′	As *E*°, except {H^+^} = 10^−7^ (*pH* = 7)
*i*	Electrical current density
*I*	Electrical current
*m*_Bio_	Biomass
*m*_Min_	Mass of precipitated minerals
MIC	Microbially influenced (usually anaerobic) corrosion (of iron)
*n*_ΔFe(0)_	Loss of metallic iron
*n*_Fe(II)_	Amount of (total) ferrous iron formed
*n*_FeAnab_	Amount of iron oxidized by the anabolism (cell synthesis)
*n*_FeCatab_	Amount of iron oxidized by the catabolism (sulfate reduction)
*n*_FeS_	Amount of ferrous sulfide (≡ *n*_SR_)
*n*_SR_	Amount of sulfate reduced (≡ *n*_FeS_)
*q*_Anab_	Partition of anabolic in total (anabolic + catabolic) electron consumption
*q^m^*_Bio_	Proportion (by mass) of biomass in corrosion crust
*q*_EMIC_	Contribution (partition) of EMIC to total corrosion by SRB
*v*	Here: voltage (otherwise volume)
*Y*_Anab_	Anabolic yield coefficient: cell mass per amount of anabolically oxidized iron

## Supporting information

**Fig. S1.** Kinetic aspects of the abiotic reaction of iron in circumneutral water, and direct (lithotrophic) iron corrosion by SRB. Availability of H^+^ ions at the metal surface and combination of adsorbed H-atoms to adsorbed H_2_ are assumed to be rate-controlling steps (‘bottle necks’), thus also controlling liberation of H_2_ into water (Bockris and Reddy, 1970; Hamann *et al*., 2007). H_2_ consumption by SRB behind the bottle neck is therefore unlikely to promote iron dissolution. Direct consumption of electrons can oxidize the iron much faster. Thickness of arrows symbolizes speed. The net reaction is always 4Fe^0^ + SO_4_^2−^ +4H_2_O → FeS + 3Fe^2+^ +8HO^−^.

**Fig. S2.** Excluding disappearance of sulfate due to coprecipitation in the corrosion crust. Grown cultures were stepwise acidified with HCl until formed corrosion products were completely dissolved. Sulfate concentration of medium did not increase.

A. Culture of strain IS4.

B. Culture of strain IS5.

C. Control culture of strain IS4 which was not acidified.

**Fig. S3.** Abiotic anaerobic iron corrosion in sterile synthetic seawater medium.

A. Production of ‘cathodic’ hydrogen by reduction of H^+^-ions (Fig. S1), and sulfide that could be formed by subsequent H_2_ utilization by SRB (4H_2_ + SO_4_^2−^ + 2H^+^ → H_2_S + 4H_2_O).

B. Original iron specimen (day 0), specimen with precipitate after 5 months (original), and after removal of precipitate (using HCl-hexamine).

**Fig. S4.** Insensitivity of non-corrosive control strain HS3 towards Fe^2+^. Addition of H_2_ to the culture including iron granules leads to rapid sulfide production (measured as sulfate loss).

**Fig. S5.** Electro-technical scheme with approximate voltage drops of the split-coupon incubation device for conductance measurement of the biogenic corrosion crust formed on corroding iron. The device circumvents interference by the noticeable contact resistance between the iron wire and the iron coupon inside the incubated bottle (Fig. 3A). The plot in the lower part depicts the voltage drop along current flow. The outer voltage (*V*_o_) is supplied and adjusted such that the voltage across the split (*V*_s_) is kept at 0.20 V while the current (*I*) is being measured. The adjusted low voltage for measurement avoids electrolysis. Measurement of *V*_s_ is carried out with a high-resistance voltmeter. *V*_c1_ and *V*_c4_ are the voltage drops due to contact resistance between the iron wire and the iron coupon (around 1 Ω), and *V*_c2_ and *V*_c3_ the arbitrarily assumed voltage drops due to the contact resistance between iron and the sulfidic crust. Voltage drop along the iron wire and the iron coupons is negligible (resistance by two and four orders or magnitude lower, respectively, than resistance of wire-coupon contact and the crust).

**Fig. S6.** Pustule (elevated precipitate) with early stage of microchimney formation above an anodic site in a culture of strain IS4 after three months of incubation. Bar, 50μm.

**Fig. S7.** Early stage of microchimney formation above an anodic area in a culture of strain IS4 after three months of incubation. Bar, 10 μm.

**Fig. S8.** Late stage of microchimney formation in a culture of strain IS4 after six months of incubation. Bar, 200μm.

**Fig. S9.** Piston electrode set-up for measurement of conductivity of a compressed siderite mineral pill.

**Fig. S10.** Sulfide production (determined as sulfate consumption) and decrease of dissolved ferrous iron due to carbonate precipitation in long-term incubations of corrosive SRB. Strain IS4 (A) which was more alkali-tolerant than strain IS5 (B) grew up to higher *pH* (*pH* increase due to Eq. 5) thus promoting precipitation according to Fe^2+^ + HO^−^ + HCO_3_^−^ → FeCO_3_ + H_2_O. This favoured formation of microchimneys (Fig. 5C). Six cultures of each strain were incubated in parallel and sacrificed at different time points for SEM analysis (Fig. 4, Fig. S6–S8). Formation of crater- and chimney-like structures in cultures of strain IS4 coincided with the drop of [Fe^2+^(aq)] below detection limit (0.2 mg l^−1^). The initial *pH* was 7.3. Strain IS4 reached *pH* ≈ 9. Activity of strain IS5 ceased at *pH* ≈ 8.

**Fig. S11.** Electron flow from metallic iron into the catabolism and anabolism.

**Table S1.** Compilation of corrosion rates recorded for (anoxic) natural and engineered environments, and for laboratory cultures of sulfate-reducing bacteria.

**Table S2.** Vitamins in used media.

**Table S3.** Conductivity values measured in an incubation device with split coupon with corrosive cultures of strains IS4 and IS5, and with sterile artificial seawater. Iron is provided as the sole source of electrons.

**Table S4.** Electrical conductivity of selected substances.

**Appendix S1.** Data, calculations and combination of Fig. S1–11 and Tables S1–4.
