# Supplementary material for: Marine sulfate-reducing bacteria cause serious corrosion of iron under electroconductive biogenic mineral crust
Source: Environ Microbiol. 2012 Jul;14(7):1772–87. doi: 10.1111/j.1462-2920.2012.02778.x (PMC3429863; doi:10.1111/j.1462-2920.2012.02778.x)
Supplement: Supplementary file 1 [file emi0014-1772-SD1.pdf]

Marine sulfate-reducing bacteria cause serious corrosion of iron under electroconductive biogenic mineral crust

Dennis Enning, Hendrik Venzlaff, Julia Garrelfs, Hang T. Dinh, Volker Meyer, Karl Mayrhofer, Achim W. Hassel, Martin Stratmann and Friedrich Widdel

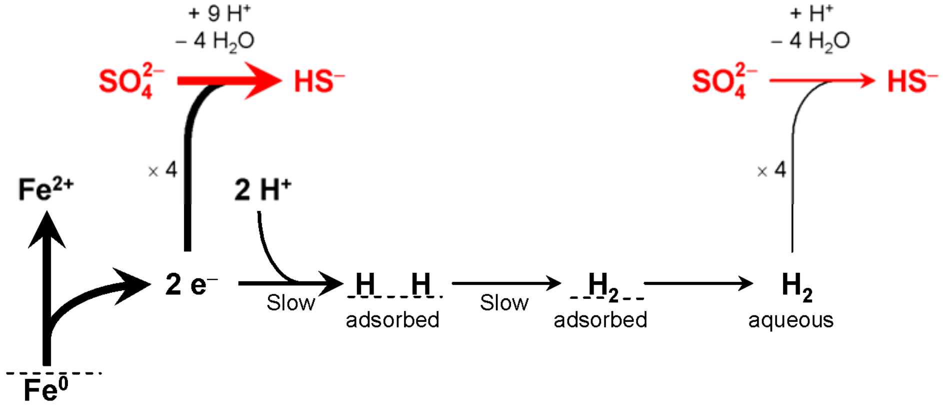

Fig. S1. Kinetic aspects of the abiotic reaction of iron in circumneutral water, and direct (lithotrophic) iron corrosion by SRB. Availability of H^+-ions at the metal surface and combination of adsorbed H-atoms to adsorbed H_2 are assumed to be rate-controlling steps ('bottle necks'), thus also controlling liberation of H_2 into water (Bockris and Reddy, 1970; Hamann *et al.*, 2007). H_2 consumption by SRB behind the bottle neck is therefore unlikely to promote iron dissolution. Direct consumption of electrons can oxidize the iron much faster. Thickness of arrows symbolizes speed. The net reaction is always $4Fe^0 + SO_4^{2-} + 4H_2O \rightarrow FeS + 3Fe^{2+} + 8OH^-$.
