# Supplementary material for: Marine sulfate-reducing bacteria cause serious corrosion of iron under electroconductive biogenic mineral crust
Source: Environ Microbiol. 2012 Jul;14(7):1772–87. doi: 10.1111/j.1462-2920.2012.02778.x (PMC3429863; doi:10.1111/j.1462-2920.2012.02778.x)
Supplement: Supplementary file 2 [file emi0014-1772-SD2.pdf]

Marine sulfate-reducing bacteria cause serious corrosion of iron under electroconductive biogenic mineral crust

Dennis Enning, Hendrik Venzlaff, Julia Garrelfs, Hang T. Dinh, Volker Meyer, Karl Mayrhofer, Achim W. Hassel, Martin Stratmann and Friedrich Widdel

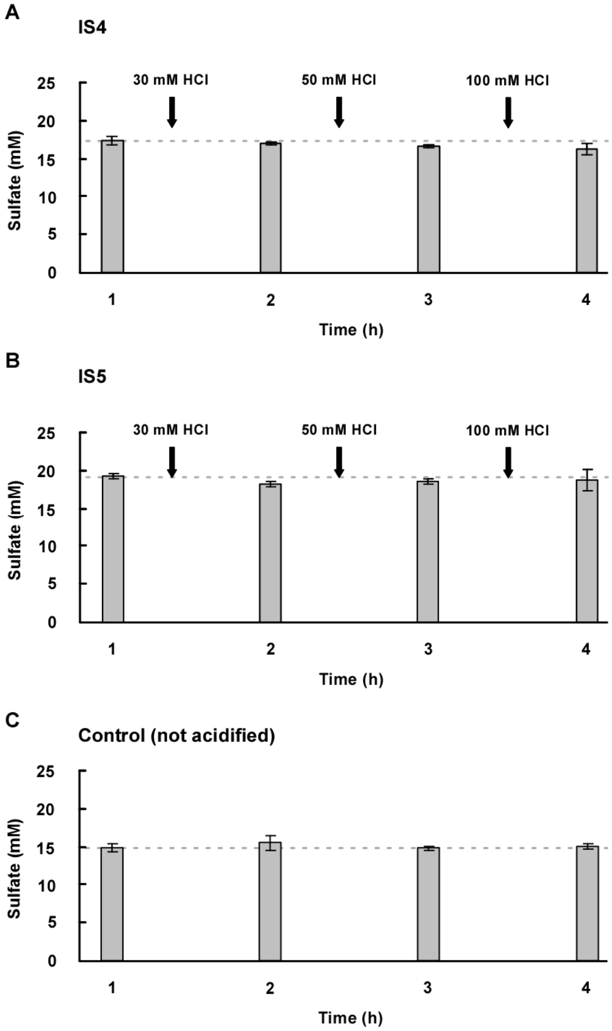

Fig. S2. Excluding disappearance of sulfate due to co-precipitation in the corrosion crust. Grown cultures were step-wise acidified with HCl until formed corrosion products were completely dissolved. Sulfate concentration of medium did not increase.
A. Culture of strain IS4.
B. Culture of strain IS5.
C. Control culture of strain IS4 which was not acidified.
