# Supplementary material for: Marine sulfate-reducing bacteria cause serious corrosion of iron under electroconductive biogenic mineral crust
Source: Environ Microbiol. 2012 Jul;14(7):1772–87. doi: 10.1111/j.1462-2920.2012.02778.x (PMC3429863; doi:10.1111/j.1462-2920.2012.02778.x)
Supplement: Supplementary file 3 [file emi0014-1772-SD3.pdf]

Marine sulfate-reducing bacteria cause serious corrosion of iron under electroconductive biogenic mineral crust

Dennis Enning, Hendrik Venzlaff, Julia Garrelfs, Hang T. Dinh, Volker Meyer, Karl Mayrhofer, Achim W. Hassel, Martin Stratmann and Friedrich Widdel

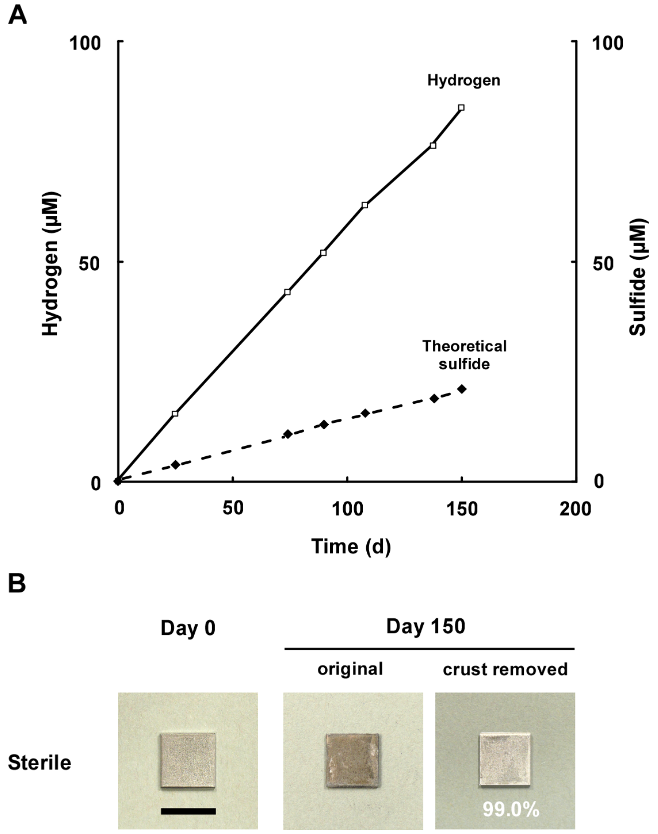

Fig. S3. Abiotic anaerobic iron corrosion in sterile synthetic seawater medium.
A. Production of ‘cathodic’ hydrogen by reduction of H^+ ions (Fig. S1), and sulfide that could be formed by H_2 utilization by SRB ($4\ H_2 + SO_4^{2-} + 2\ H^+ \rightarrow H_2S + 4\ H_2O$).
B. Original iron specimen (day 0), specimen with precipitate after 5 months (original) and after removal of precipitate (using HCl-hexamine).
