# Supplementary material for: Marine sulfate-reducing bacteria cause serious corrosion of iron under electroconductive biogenic mineral crust
Source: Environ Microbiol. 2012 Jul;14(7):1772–87. doi: 10.1111/j.1462-2920.2012.02778.x (PMC3429863; doi:10.1111/j.1462-2920.2012.02778.x)
Supplement: Supplementary file 5 [file emi0014-1772-SD5.pdf]

Marine sulfate-reducing bacteria cause serious corrosion of iron under electroconductive biogenic mineral crust

Dennis Enning, Hendrik Venzlaff, Julia Garrelfs, Hang T. Dinh, Volker Meyer, Karl Mayrhofer, Achim W. Hassel, Martin Stratmann and Friedrich Widdel

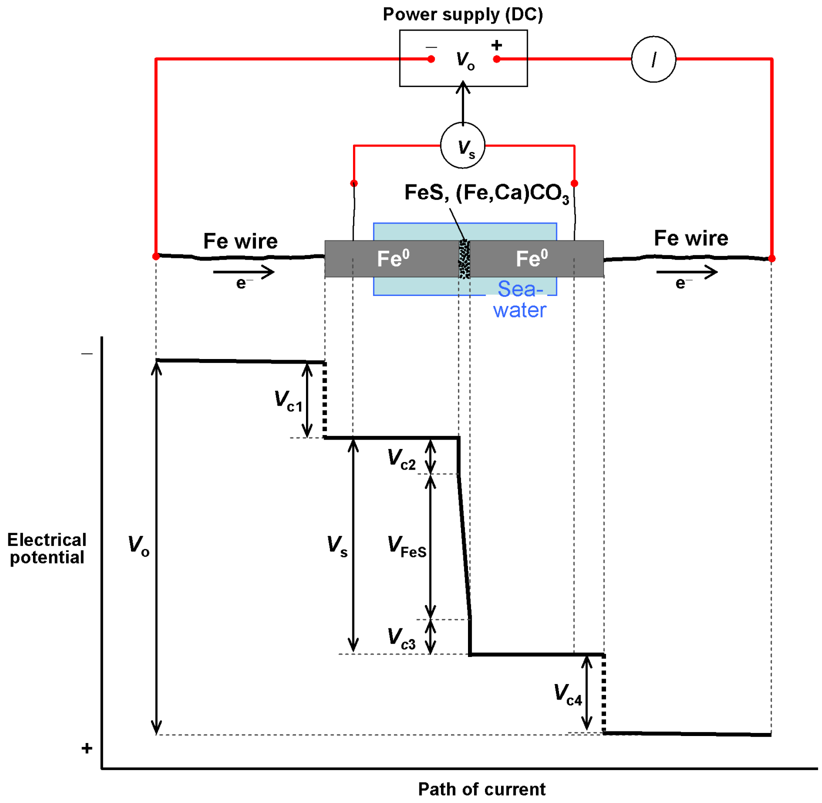

Fig. S5. Electro-technical scheme with approximate voltage drops of the split-coupon incubation device for conductance measurement of the biogenic crust formed on corroding iron. The device circumvents interference by the noticeable contact resistance between the iron wire and the iron coupon inside the incubated bottle (Fig. 3A). The plot in the lower part depicts the voltage drop along current flow. The outer voltage (V_o) is supplied and adjusted such that the voltage across the split (V_s) is kept at 0.20 V while the current (I) is being measured. The adjusted low voltage for measurement avoids electrolysis. Measurement of V_s is carried out with a high-resistance voltmeter. V_{c1} and V_{c4} are the voltage drops due to contact resistance between the iron wire and the iron coupon (around 1 Ω), and V_{c2} and V_{c3} the arbitrarily assumed voltage drops due to the contact resistance between iron and the sulfidic crust. Voltage drop along the iron wire and the iron coupons is negligible (resistance by two and four orders or magnitude lower, respectively, than resistance of wire-coupon contact and the crust).
