# Supplementary material for: Marine sulfate-reducing bacteria cause serious corrosion of iron under electroconductive biogenic mineral crust
Source: Environ Microbiol. 2012 Jul;14(7):1772–87. doi: 10.1111/j.1462-2920.2012.02778.x (PMC3429863; doi:10.1111/j.1462-2920.2012.02778.x)
Supplement: Supplementary file 6 [file emi0014-1772-SD6.pdf]

Marine sulfate-reducing bacteria cause serious corrosion of iron under electroconductive biogenic mineral crust

Dennis Enning, Hendrik Venzlaff, Julia Garrelfs, Hang T. Dinh, Volker Meyer, Karl Mayrhofer, Achim W. Hassel, Martin Stratmann and Friedrich Widdel

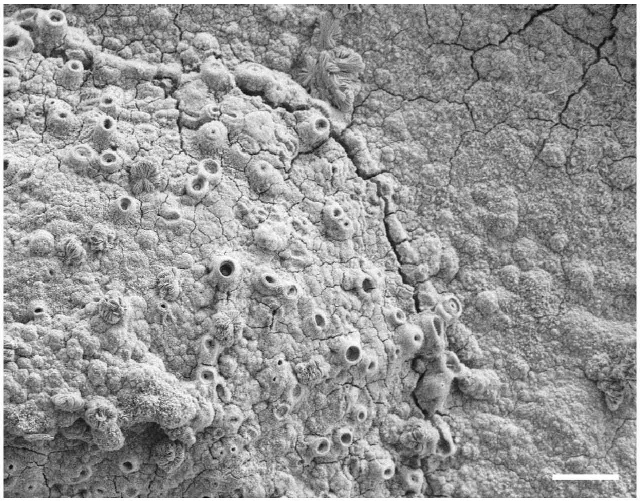

Fig. S6. Pustule (elevated precipitate) with early stage of micro-chimney formation above an anodic site in a culture of strain IS4 after three months of incubation. Bar, 50 µm.
