# Supplementary material for: Marine sulfate-reducing bacteria cause serious corrosion of iron under electroconductive biogenic mineral crust
Source: Environ Microbiol. 2012 Jul;14(7):1772–87. doi: 10.1111/j.1462-2920.2012.02778.x (PMC3429863; doi:10.1111/j.1462-2920.2012.02778.x)
Supplement: Supplementary file 10 [file emi0014-1772-SD10.pdf]

Marine sulfate-reducing bacteria cause serious corrosion of iron under electroconductive biogenic mineral crust

Dennis Enning, Hendrik Venzlaff, Julia Garrelfs, Hang T. Dinh, Volker Meyer, Karl Mayrhofer, Achim W. Hassel, Martin Stratmann and Friedrich Widdel

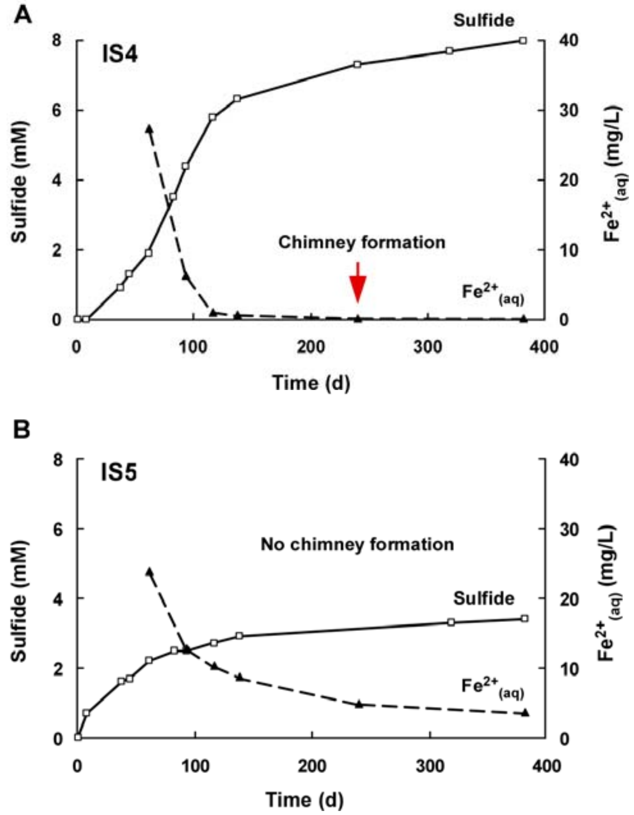

Fig. S10. Sulfide production (determined as sulfate consumption) and decrease of dissolved ferrous iron due to carbonate precipitation in long-term incubations of corrosive SRB. Strain IS4 (**A**) which was more alkali-tolerant than strain IS5 (**B**) grew up to higher *pH* [*pH* increase due to equation (5)] thus promoting precipitation according to $Fe^{2+} + HO^{-} + HCO_3^{-} \rightarrow FeCO_3 + H_2O$. This favored formation of micro-chimneys (Fig. 5C). Six cultures of each strain were incubated in parallel and sacrificed at different time points for SEM analysis (Fig. 4, Figs S6 to 8). Formation of crater- and chimney-like structures in cultures of strain IS4 coincided with the drop of [$Fe^{2+}_{(aq)}$] below detection limit (0.2 mg/l). The initial *pH* was 7.3. Strain IS4 reached *pH* ≈ 9. Activity of strain IS5 ceased at *pH* ≈ 8.
