# Supplementary material for: Marine sulfate-reducing bacteria cause serious corrosion of iron under electroconductive biogenic mineral crust
Source: Environ Microbiol. 2012 Jul;14(7):1772–87. doi: 10.1111/j.1462-2920.2012.02778.x (PMC3429863; doi:10.1111/j.1462-2920.2012.02778.x)
Supplement: Supplementary file 12 [file emi0014-1772-SD12.pdf]

Marine sulfate-reducing bacteria cause serious corrosion of iron under electroconductive biogenic mineral crust

Dennis Enning, Hendrik Venzlaff, Julia Garrelfs, Hang T. Dinh, Volker Meyer, Karl Mayrhofer, Achim W. Hassel, Martin Stratmann and Friedrich Widdel

Table S1. Compilation of corrosion rates recorded for (anoxic) natural and engineered environments, and for laboratory cultures of sulfate-reducing bacteria.

**Location**	**Type of environment**	**Corrosion rate (mm yr^{-1})** [a]	**Method** [b]	**Reference**
Bohai Bay, China	Marine sediment	**0.03 – 0.09**	Weight loss	Li (2009)
Draugen Oil Field, Norwegian North Sea	Produced water re-injection system; untreated produced water	**0.12**	Electrochemical [c]	Vik *et al.* (2007)
Tonnenlegerbucht, German North Sea	Marine anoxic sediment	**0.12 – 0.28**	Weight loss	This study
South Korea	Gas transmission pipelines; disbonded coating	**0.33 – 0.47**	Pit measurement	Li *et al.* (2000)
Gullfaks Oil Field, Norwegian North Sea	Water injection system; biocide treatment	**0.09 – 0.75**	Weight loss	Boedtker *et al.* (2008)
**Culture**	**Type of energy source, medium** [d]	**Corrosion rate (mm yr^{-1})** [e]	**Method**	**Reference**
Desulfovibrio sp.	Lactate-based, freshwater	**0.007**	Weight loss	Hardy and Brown (1984)
Desulfovibrio vulgaris, Woolwich	Lactate-based, freshwater	**0.024**	Weight loss	Gaylarde (1992)
Desulfovibrio desulfuricans, New Jersey	Lactate-based, marine	**0.094**	Weight loss	Beech *et al.* (1994)
Desulfovibrio desulfuricans	Lactate-based, brackish	**0.285**	Weight loss	Bell and Lim (1981)
SRB bioreactor	Lactate-based, brackish	**0.408**	Weight loss	Hubert *et al.* (2005)
Desulfovibrio vulgaris, Hildenborough	H_2, freshwater [f]	**0.500**	Electrochemical [g]	Pankhania *et al.* (1986)
Sterile artificial seawater	Fe^0 lithotrophy, marine	**0.010**	Weight loss	This study
Desulfopila inferna	Fe^0 lithotrophy, marine	**0.012**	Weight loss	This study
'Desulfopila corrodens', strain IS4	Fe^0 lithotrophy, marine	**0.311**	Weight loss	This study
'Desulfovibrio ferrophilus', strain IS5	Fe^0 lithotrophy, marine	**0.710**	Weight loss	This study

a. Corrosion rate in natural and engineered environments. The recorded range is given. Corrosion must not necessarily be influenced by SRB in these systems.
b. Method of corrosion rate determination. Weight loss is considered the most accurate technique.
c. A linear polarisation resistance (LPR) probe was used.
d. Energy source for SRB metabolism is indicated. All cultures contained iron or mild steel. 'Fe^0 lithotrophy' indicates that metallic iron was the only available source of reducing equivalents. Salt content of media (freshwater, brackish, marine) is also designated.
e. Corrosion rate in cultures of SRB. The maximal reported value is given. The large range of corrosion rates for SRB with external energy source is attributed to the sometimes protective properties of formed FeS films (see text).
f. H_2 from previous polarisation experiment was available in these cultures.
g. Calculated from slope of cathodic polarisation curve by overvoltage-intercept method.
