# Supplementary material for: Marine sulfate-reducing bacteria cause serious corrosion of iron under electroconductive biogenic mineral crust
Source: Environ Microbiol. 2012 Jul;14(7):1772–87. doi: 10.1111/j.1462-2920.2012.02778.x (PMC3429863; doi:10.1111/j.1462-2920.2012.02778.x)
Supplement: Supplementary file 13 [file emi0014-1772-SD13.pdf]

Marine sulfate-reducing bacteria cause serious corrosion of iron under electroconductive biogenic mineral crust

Dennis Enning, Hendrik Venzlaff, Julia Garrelfs, Hang T. Dinh, Volker Meyer, Karl Mayrhofer, Achim W. Hassel, Martin Stratmann and Friedrich Widdel

Table S2. Vitamins in used media.

Vitamin	Concentration (µg l^{-1})
4-Aminobenzoic acid	40
D(+)-Biotin	10
Nicotinic acid	100
Ca-D(+)-Pantothenate	50
Pyridoxine hydrochloride	150
Folic acid	40
Liponic acid	15
Thiamine hydrochloride	100
Riboflavin	25
Cyanocobalamin	50
