# Supplementary material for: Marine sulfate-reducing bacteria cause serious corrosion of iron under electroconductive biogenic mineral crust
Source: Environ Microbiol. 2012 Jul;14(7):1772–87. doi: 10.1111/j.1462-2920.2012.02778.x (PMC3429863; doi:10.1111/j.1462-2920.2012.02778.x)
Supplement: Supplementary file 14 [file emi0014-1772-SD14.pdf]

Marine sulfate-reducing bacteria cause serious corrosion of iron under electroconductive biogenic mineral crust

Dennis Enning, Hendrik Venzlaff, Julia Garrelfs, Hang T. Dinh, Volker Meyer, Karl Mayrhofer, Achim W. Hassel, Martin Stratmann and Friedrich Widdel

Table S3. Conductivity values measured in an incubation device with split coupon with corrosive cultures of strains IS4 and IS5, and with sterile artificial seawater. Iron is provided as the sole source of electrons.

	Slot bridged by sulphidic crust [a]	Slot bridged by seawater (control) [b]
**Supplied outer voltage (V_O)**	0.2 – 0.4 V	0.2 – 0.3 V
**Voltage at split coupon (V_S)**	0.20 V	0.20 V
**Measured current**	0.14 – 0.31 A	$0.3 – 0.7 \cdot 10^{-4}$ A
**Conductance**	0.72 – 1.56 S	$1.5 – 3.5 \cdot 10^{-4}$ S
**Conductivity**	27.4 – 43.5 S m^{-1}	$0.5 – 1.7 \cdot 10^{-2}$ S m^{-1}
**Resistance**	0.64 – 1.39 Ω	$2.9 – 6.7 \cdot 10^{3}$ Ω
**Resistivity**	$2.3 – 3.7 \cdot 10^{-2}$ Ω m	$0.6 – 2.1 \cdot 10^{2}$ Ω m

a. Values refer to data range obtained from 4 biological replicates (3 cultures of strain IS4 and one culture of strain IS5);
b. Values refer to data range obtained from 3 sterile incubations.
