# Supplementary material for: Marine sulfate-reducing bacteria cause serious corrosion of iron under electroconductive biogenic mineral crust
Source: Environ Microbiol. 2012 Jul;14(7):1772–87. doi: 10.1111/j.1462-2920.2012.02778.x (PMC3429863; doi:10.1111/j.1462-2920.2012.02778.x)
Supplement: Supplementary file 15 [file emi0014-1772-SD15.pdf]

Marine sulfate-reducing bacteria cause serious corrosion of iron under electroconductive biogenic mineral crust

Dennis Enning, Hendrik Venzlaff, Julia Garrelfs, Hang T. Dinh, Volker Meyer, Karl Mayrhofer, Achim W. Hassel, Martin Stratmann and Friedrich Widdel

Table S4. Electrical conductivity of selected substances.

Material		Conductivity (S m^{-1})	Reference
Iron, 99.98% pure		$1.1 \cdot 10^{7}$	a
Steel, plain		$5.6 \cdot 10^{6}$	b
Graphite		$1.5 \cdot 10^{6}$	b
Steel, stainless		$1.4 \cdot 10^{6}$	b
Troilite (FeS)		$1.0 \cdot 10^{1} – 1.0 \cdot 10^{6}$	Pearce *et al.* (2006)
Pyrrhotite ($Fe_{1-X}S$), mineral		$2.0 \cdot 10^{4} – 1.0 \cdot 10^{5}$	Parasnis (1956)
Pyrrhotite ($Fe_{1-X}S$), ore		$1.0 \cdot 10^{3} – 1.0 \cdot 10^{5}$	Parasnis (1956)
Magnetite (Fe_3O_4)		$1.0 \cdot 10^{4} – 1.0 \cdot 10^{5}$	Schwertmann & Cornell (2003)
Pyrite (FeS_2), mineral		$2.0 \cdot 10^{1} – 2.0 \cdot 10^{4}$	Parasnis (1956)
Pyrite (FeS_2), ore		$1.0 \cdot 10^{-1} – 1.0 \cdot 10^{4}$	Parasnis (1956)
**SRB corrosion crust**		**$2.7 \cdot 10^{1} – 6.4 \cdot 10^{1}$**	**This study**
Germanium		$2.2 \cdot 10^{0}$	b
G. sulfurreducens biofilm		$0.5 \cdot 10^{0}$	Malvenkar et al. (2011)
Silicon		$1.6 \cdot 10^{-3}$	b
**Siderite mineral**		**$1.2 \cdot 10^{-7}$**	**This study**
Goethite (FeOOH)	approx.	$1.0 \cdot 10^{-7}$	Schwertmann & Cornell (2003)
Siderite ($FeCO_3$)		$1.2 \cdot 10^{-10}$	Schön (1996)
Calcite ($CaCO_3$)		$2.0 \cdot 10^{-13} – 1.1 \cdot 10^{-14}$	Schön (1996)

a. CRC Handbook of Chemistry and Physics.
b. www.physics.info/electric-resistance/
