# Supplementary material for: Marine sulfate-reducing bacteria cause serious corrosion of iron under electroconductive biogenic mineral crust
Source: Environ Microbiol. 2012 Jul;14(7):1772–87. doi: 10.1111/j.1462-2920.2012.02778.x (PMC3429863; doi:10.1111/j.1462-2920.2012.02778.x)
Supplement: Supplementary file 16 [file emi0014-1772-SD16.pdf]

# Marine sulfate-reducing bacteria cause serious corrosion of iron under electroconductive biogenic mineral crust

**Dennis Enning, Hendrik Venzlaff, Julia Garrelfs, Hang T. Dinh, Volker Meyer, Karl Mayrhofer, Achim W. Hassel, Martin Stratmann and Friedrich Widdel**

## Appendix S1

### *Costs due to iron corrosion (aerobic, anaerobic)*

Several studies have addressed the costs due to metal (iron + other) corrosion, the most comprehensive ones providing data for the United States (Revie, 2011). Here, a recent report (Koch *et al*., 2001) provided annual direct costs due to metal corrosion of $276 \cdot 10^9$ \$, which is 3.1% of U.S. GDP. 'Indirect' costs to the user were conservatively estimated to be similar, so that total costs due to corrosion to society may be as high as 6% of GDP. Similarly, costs of metallic corrosion in other developed countries have been estimated to range between 2 and 3% of GNP [see Revie (2011) for review]. As iron is the by far most widely used metal and particularly prone to corrosion (oxidation crusts of iron provide far less protection than those of other metals), the calculated costs due to metal corrosion are largely those of iron corrosion. Even though aerobic (abiotic) rusting is the most common type of iron corrosion, anaerobic corrosion by microbial activity is a dominant type in particular industrial water systems in the production and transportation of oil, gas and energy carriers (Lee *et al*., 1995; Jack, 2002; Beech and Sunner, 2007).

### *Free energy values and redox potentials of dissolved iron*

Reactions involving dissolved ferrous iron were based on a revised $\Delta G_f°$-value of $Fe^{2+}$ (aq) of –90.53 kJ mol$^{-1}$ (Rickard and Luther, 2007), the corresponding $E°$ ($Fe^{2+}/Fe^0$) value being –0.469 V. Other commonly used corresponding values are –84.9 kJ mol$^{-1}$ and –0.440 V (Randall and Frandsen, 1932; Dean, 1992; Dinh *et al*., 2004; Atkins and De Paula, 2006). The least negative corresponding values are –78.87 kJ mol$^{-1}$ and –0.409 V (Patrick and Thompson, 1952).

### *Indirect corrosion (CMIC) of iron by sulfate-reducing bacteria*

Hydrogen sulfide from sulfate reduction with biomass-derived organic carbon (here simplified as carbohydrate ⟨$CH_2O$⟩ or carbohydrate-building unit ⟨HCOH⟩) according to

$$2\,\langle HCOH\rangle + SO_4^{2-} \rightarrow 2\,HCO_3^- + H_2S \qquad (S1)$$

attacks metallic iron in an abiotic reaction [see also (Dinh *et al*., 2004)]:

$$Fe^0 + H_2S \rightarrow FeS + H_2. \qquad (S2)$$

The sum reaction is

$$2\,\langle HCOH\rangle + SO_4^{2-} + Fe^0 \rightarrow 2\,HCO_3^- + FeS + H_2. \qquad (S3)$$

$H_2$ from equation (S2) as an excellent electron donor for SRB again leads to $H_2S$ according to

$$H_2 + \tfrac{1}{4}\,SO_4^{2-} + \tfrac{1}{2}\,H^+ \rightarrow \tfrac{1}{4}\,H_2S + H_2O, \qquad (S4)$$

again attacking ¼ $Fe^0$ (Eq. S2 divided by 4). The sum is now

$$2\,\langle HCOH\rangle + 1\tfrac{1}{4}\,SO_4^{2-} + 1\tfrac{1}{4}\,Fe^0 + \tfrac{1}{2}\,H^+$$

$$\rightarrow 2\,HCO_3^- + 1\tfrac{1}{4}\,FeS + \tfrac{1}{4}\,H_2 + H_2O \qquad (S5)$$

Reduction of ¼ $SO_4^{2-}$ with ¼ $H_2$ yields $^1/_{16}$ $H_2S$ attacking $^1/_{16}$ $Fe^0$. Continuing *ad infinitum* and summing yields

$$2\,\langle HCOH\rangle + \left[\sum_{n=0}^{\infty}\left(\frac{1}{4}\right)^n\right] SO_4^{2-} + \left[\sum_{n=0}^{\infty}\left(\frac{1}{4}\right)^n\right] Fe^0 + \left[\frac{1}{2}\sum_{n=0}^{\infty}\left(\frac{1}{4}\right)^n\right] H^+$$
$$\longrightarrow 2\,HCO_3^- + \left[\sum_{n=0}^{\infty}\left(\frac{1}{4}\right)^n\right] FeS + \left[\sum_{n=0}^{\infty}\left(\frac{1}{4}\right)^n\right] H_2O \qquad (S6)$$

The geometric series converges according to

$$\sum_{n=0}^{\infty}\left(\frac{1}{4}\right)^n = \frac{1-\left(\tfrac{1}{4}\right)^{\infty}}{1-\left(\tfrac{1}{4}\right)} = \frac{4}{3}\ , \qquad (S7)$$

thus converting equation (S5) to

$$2\,\langle HCOH\rangle + {}^4/_3\,SO_4^{2-} + {}^4/_3\,Fe^0 + {}^2/_3\,H^+ \rightarrow 2\,HCO_3^- + {}^4/_3\,FeS + {}^4/_3\,H_2O\ . \qquad (S8)$$

Multiplying by $^3/_2$ so as to avoid fractional stoichiometry yields

$$3\,\langle HCOH\rangle + 2\,SO_4^{2-} + 2\,Fe^0 + H^+ \rightarrow 3\,HCO_3^- + 2\,FeS + 2\,H_2O\ . \qquad (S9)$$

Note that a mere co-oxidation of ⟨HCOH⟩ and $Fe^0$ can result in the same equation.

*Metal loss rates*

The rate (velocity) of the loss of metal mass ($m$) by corrosion is $v_{corr}^{\theta}$ = d$m$/d$t$ or, if constant during an experimental time interval $\Delta t$, also $v_{corr}^{\theta} = \Delta m/\Delta t$. Division by density, $\rho$, and surface area, $a$, yields the thickness ($\theta$) loss rate, $v_{corr}^{\theta} = \Delta\theta/\Delta t = \Delta m/(a\,\rho\,\Delta t)$. With SI units and $\rho$ = 7.87 kg m$^{-3}$ (mild steel EN 1.0330), the thickness loss rate is

$$v_{corr}^{\theta}\ (\mathrm{m\ s^{-1}}) = 0.1271\ (\mathrm{m^3\ kg^{-1}})\ \frac{\Delta m}{a\,\Delta t} \qquad (S10)$$

If, for convenience, thickness is measured in mm, mass in mg, area in cm$^{-2}$, and time in yr, the thickness loss rate is

$$v_{corr}^{\theta}\ (\mathrm{mm\ yr^{-1}}) = 1.27\cdot 10^{-3}\ (\mathrm{mm\ mg^{-1}\ cm^2})\ \frac{\Delta m}{a\,\Delta t} \qquad (S11)$$

*Corrosion current density*

The rate (velocity) of the loss of metal amount ($n$, in mol) by corrosion is derived from mass ($m$) loss (see above) as $v^n_{\mathrm{corr}} = \Delta m/(M_a\, \Delta t)$, with $M_a$ being the atomic mass. With $n_e$ as the number of electrons released per metal atom and the Faraday constant $F$, the current of electron loss is $i_{\mathrm{corr}} = n_e\, F\, v^n_{\mathrm{corr}} = n_e\, F\, \Delta m/(M_a\, \Delta t)$. Division by surface area, $a$, yields the current density,

$$i_{\mathrm{corr}}\ (\mathrm{A\ m^{-2}}) = \frac{n_e\, F\, \Delta m}{a\, M_a\, \Delta t}\ . \qquad \text{(S12)}$$

If the corrosion rate is given as thickness ($\theta$) loss instead of mass loss, the density, $\rho$, must be included:

$$i_{\mathrm{corr}}\ (\mathrm{A\ m^{-2}}) = \frac{n_e\, F\, \rho\, \Delta\theta}{M_a\, \Delta t}\ . \qquad \text{(S13)}$$

With SI units, $n_e = 2$, $M_a = 55.85 \cdot 10^{-3}$ kg mol$^{-1}$ (for iron) and $\rho = 7.87 \cdot 10^{3}$ kg m$^{-3}$ (mild steel EN 1.0330), and $F = 96{,}485$ C mol$^{-1}$, formulas (S12) and (S13) convert to

$$i_{\mathrm{corr}}\ (\mathrm{A\ m^{-2}}) = 3.455 \cdot 10^{6}\ (\mathrm{A\ s\ kg^{-1}})\ \frac{\Delta m}{a\, \Delta t} \qquad \text{(S14)}$$

$$i_{\mathrm{corr}}\ (\mathrm{A\ m^{-2}}) = 2.72 \cdot 10^{10}\ (\mathrm{A\ m^{-3}\ s})\ \frac{\Delta\theta}{\Delta t} \qquad \text{(S15)}$$

If, for convenience, thickness is measured in mm, mass in mg, area in cm$^2$, and time in yr, the respective corrosion current density is

$$i_{\mathrm{corr}}\ (\mathrm{A\ cm^{-2}}) = 1.095 \cdot 10^{-7}\ (\mathrm{A\ yr\ mg^{-1}})\ \frac{\Delta m}{a\, \Delta t} \qquad \text{(S16)}$$

$$i_{\mathrm{corr}}\ (\mathrm{A\ cm^{-2}}) = 8.62 \cdot 10^{-5}\ (\mathrm{A\ yr\ cm^{-2}\ mm^{-1}})\ \frac{\Delta\theta}{\Delta t}\ (\text{fraction in mm yr}^{-1}) \qquad \text{(S17)}$$

*Crust conductivity and redox potential difference*

Electrical current, $I$, through a cylindrical or cubical body of ohmic behavior is proportional to the applied voltage, $V$, and area perpendicular to current direction, $a$, and inversely proportional to length, $d$, i.e. $I = \sigma\, V\, a\, /\, d$. The proportionality constant, $\sigma$, is the specific conductance or conductivity (A V$^{-1}$ m$^{-1}$; S m$^{-1}$, $\Omega^{-1}$ m$^{-1}$). The (argument of the vectorial) electrical field strength sustaining the current $I$ is thus $V/d = I/\sigma a$. With current density $i = I/a$ we can write

$$\frac{V}{d} = \frac{i}{\sigma} \qquad \text{(S18)}$$

For a corrosion current of $i_{\mathrm{corr}} = 0.61$ A m$^{-2}$ (calculated from loss of $Fe^0$) and the determined conductivity of $\sigma = 50$ S m$^{-1}$, the field strength between metal and colonized crust would be only

$$\frac{V}{d} = 1.2 \cdot 10^{-2}\ \mathrm{V\ m^{-1}}, \quad \text{(S19)}$$

which is as low as $1.2 \cdot 10^{-4}$ V across a crust with a realistic thickness of 1 cm. The voltage $V$ is the difference between the operational redox potentials of the electron-accepting sulfate reduction (SR) and the electron-donating iron dissolution (FeDiss), $V = \Delta\phi = \phi_{\mathrm{SR}} - \phi_{\mathrm{FeDiss}}$. Due to reaction overpotentials, $\Delta\phi$ must be within the difference of the equilibrium redox potentials ($E$), i.e. $V = \Delta\phi < \Delta E = E_{\mathrm{SR}} - E_{\mathrm{FeDiss}}$. $E_{\mathrm{SR}}$ and $E_{\mathrm{FeDiss}}$ are calculated from the half reactions and free energies of formation[1] (Thauer *et al.*, 1977; Garrels and Christ, 1985), first for standard conditions at $pH$ = 7 ($E°'$, here used for $E°_{pH7}$), and subsequently for near-real conditions assuming activities (in seawater) of $\{SO_4^{2-}\} = 3 \cdot 10^{-3}$ and $\{HCO_3^-\} = 1.5 \cdot 10^{-2}$ [from applied concentrations and activity coefficients (Stumm and Morgan, 1996)] and $pH$ = 8.

$$SO_4^{2-} + FeCO_3 + 9\ H^+ + 8\ e^- \rightleftarrows FeS + HCO_3^- + 4\ H_2O \quad \text{(S20)}$$

$E°'_{\mathrm{SR}} = -0.175$ V;   $E_{\mathrm{SR}} = -0.25$ V

$$Fe^0 + HCO_3^- \rightleftarrows FeCO_3 + H^+ + 2\ e^- \quad \text{(S21)}$$

$E°'_{\mathrm{FeDiss}} = -0.62$ V;   $E_{\mathrm{FeDiss}} = -0.60$ V

The resulting $\Delta E = -0.35$ V gives more than sufficient leeway for a 'self-adjusting' $\Delta\phi$ during (the irreversible) electron withdrawal by corrosive SRB.

*Acidity of $Fe^{2+}$*

The $pK_a$ of an acid, viz. a reaction (acid ⇄ base + proton) with equilibrium constant $K_{eq}$ (also termed $K_a$) is

$$pK_a = -\lg K_a = -\lg K_{eq} = -(\lg e) \ln K_{eq} = -(\lg e)\frac{-\Delta G^\circ_{\rightarrow}}{RT} = 0.4343\frac{\Delta G^\circ_{\rightarrow}}{RT}\ . \quad \text{(S22)}$$

$\Delta G°_{\rightarrow}$ is the standard free energy of the forward reaction. Hydrated $Fe^{2+}$ ions tend to release a proton from the water shell according to

$$[Fe(OH_2)_6]^{2+} \rightleftarrows [Fe(OH_2)_5(OH)]^+ + H^+, \quad \text{(S23)}$$

which in thermodynamic data compilations and for calculations is usually simplified as

$$Fe^{2+} + H_2O \rightleftarrows Fe(OH)^+ + H^+ \quad \text{(S24)}$$

$$K_{eq} = \left(\frac{\{Fe(OH)^+\}\ \{H^+\}}{\{Fe^{2+}\}\ \{H_2O\}}\right)_{eq}$$

Free energy with revised $\Delta G_f°$–value (Rickard and Luther, 2007) for $Fe^{2+}$ of −90.5 kJ mol$^{-1}$ is $\Delta G°_{\rightarrow} = +50.4$ kJ mol$^{-1}$, yielding

$$pK_a = 8.8\ \ (T = 298\ \mathrm{K}). \quad \text{(S25)}$$

---

[1] Used $\Delta G_f°$-values (kJ mol$^{-1}$): $Fe^0$, 0.00; $Fe^{2+}$, –90.53; $FeCO_3$ (c), –666.7; FeS (c), –100.4; $H^+$ ($pH$ = 7), –40.0; $HCO_3^-$, –586.8; $H_2O$, –237.18; $SO_4^{2-}$, –744.6.

Free energy with a formerly common $\Delta G_f°$-value for $Fe^{2+}$ of −78.87 kJ $mol^{-1}$ yields $\Delta G°_{\rightarrow}$ = +38.8 kJ and

$$pK_a = 6.6 \quad (T = 298\text{ K}). \tag{S26}$$

It is possible that also the $\Delta G_f°$-value for $Fe(OH)^+$ (−277.3 kJ $mol^{-1}$) requires revision (origin of present value not investigated). Hence, the $pK_a$-values calculated here may be regarded only as upper and lower limits of a range into which a fully revised $pK_a$-value will fall.

*Content of formed biomass in precipitated corrosion products*

The fraction of biomass in the precipitated corrosion products is expressed as the quotient

$$q^m{}_{\text{Bio}} = \frac{m_{\text{Bio}}}{m_{\text{Min}} + m_{\text{Bio}}}, \qquad (14, 15, S27)$$

with $m_{\text{Bio}}$ indicating the biomass and $m_{\text{Min}}$ the mineral mass (superscript '*m*' indicates mass ratio rather than molar ratio in other quotients). Because there is presently no convenient analytical method, the quotient is calculated from the amounts of iron oxidized and sulfate reduced.

The mineral mass, $m_{\text{Min}}$, is that of precipitated FeS and $FeCO_3$, and possibly co-precipitated alkaline earth (Mg + Ca, here Ae) carbonates, $AeCO_3$, i.e. $m_{\text{Min}} = m_{\text{FeS}} + m_{\text{FeCO}_3} + m_{\text{AeCO}_3}$. This is expressed via molecular masses (*M*) and amounts (*n*, mol) as $m_{\text{Min}} = M_{\text{FeS}}\, n_{\text{FeS}} + M_{\text{FeCO3}}\, n_{\text{FeCO}_3} + m_{\text{AeCO}_3}$. Because FeS precipitation scavenges all formed sulfide, $n_{\text{FeS}} = n_{\text{SR}}$, the amount of sulfate reduced. Assuming that all ferrous iron formed during EMIC and not precipitated as FeS is precipitated as $FeCO_3$, the amount of the latter is total iron loss by EMIC minus sulfidic iron, i.e. $n_{\text{FeCO}_3} = n_{\text{FeEMIC}} - n_{\text{FeS}}$ or $n_{\text{FeCO}_3} = n_{\text{FeEMIC}} - n_{\text{SR}}$. This yields for the mineral mass

$$m_{\text{Min}} = M_{\text{FeS}}\, n_{\text{SR}} + M_{\text{FeCO3}}\, (n_{\text{FeEMIC}} - n_{\text{SR}}) + m_{\text{AeCO3}} \tag{S28}$$

The biomass (e.g., in g) formed per amount (e.g., in mol) of iron used for (attributed to) the anabolism (biosynthesis) is calculated from the predicted (Eq. 11, 12) yield coefficient $Y_{\text{FeAnb}}$ (biomass per iron oxidized by the anabolism) and the amount of iron needed for the anabolism, $m_{\text{Bio}} = Y_{\text{Anab}}\, n_{\text{FeAnab}}$. Because $n_{\text{FeAnab}} = n_{\Delta\text{Fe(0)}} - 4\, n_{\text{SR}}$ (Eq. 8), the biomass is

$$m_{\text{Bio}} = Y_{\text{Anab}}\, (n_{\Delta\text{Fe(0)}} - 4\, n_{\text{SR}}). \tag{S29}$$

With equations (S28) and (S29), the quotient in equation (S27) converts to

$$q^m{}_{\text{Bio}} = \frac{Y_{\text{Anab}}(n_{\Delta\text{Fe(0)}} - 4\, n_{\text{SR}})}{M_{\text{FeS}} n_{\text{SR}} + M_{\text{FeCO}_3}(n_{\Delta\text{Fe(0)}} - n_{\text{SR}}) + m_{\text{AeCO}_3} + Y_{\text{FeAnb}}(n_{\Delta\text{Fe(0)}} - 4\, n_{\text{SR}})} \tag{S30}$$

or (with $M_{\text{FeS}}$ = 87.9 and $M_{\text{FeCO3}}$ = 115.9 g $mol^{-1}$)

$$q^m{}_{\text{Bio}} = \frac{Y_{\text{Anab}}(n_{\Delta\text{Fe(0)}} - 4 n_{\text{SR}})}{115.9\;\; n_{\Delta\text{Fe(0)}} - 28\, n_{\text{SR}} + m_{\text{AeCO}_3} + Y_{\text{Anab}}(n_{\Delta\text{Fe(0)}} - 4 n_{\text{SR}})}\,. \tag{S31}$$

If $AeCO_3$ is present but not included in equation (S27), it leads to an overestimate of $q^m{}_{Bio}$, so that

$$q^m{}_{Bio} \le \frac{Y_{Anab}(n_{\Delta Fe(0)} - 4\, n_{SR})}{115.9\, n_{\Delta Fe(0)} - 28\, n_{SR} + Y_{Anab}(n_{\Delta Fe(0)} - 4\, n_{SR})}, \quad (S32)$$

(for units g, mol). Equations (11) and (12) predict $Y_{FeAna(aut)}$ = 12.0 g mol$^{-1}$ for autotrophic and $Y_{FeAna(het)}$ = 32.2 g mol$^{-1}$, yielding

$$q^m{}_{Bio(aut)} \le \frac{n_{\Delta Fe(0)} - 4\, n_{SR}}{10.66\, n_{\Delta Fe(0)} - 6.33\, n_{SR}} \quad (S33)$$

and

$$q^m{}_{Bio(het)} \le \frac{n_{\Delta Fe(0)} - 4\, n_{SR}}{4.60\, n_{\Delta Fe(0)} - 4.87\, n_{SR}}, \quad (S34)$$

respectively.

*Simplified calculation of the contribution of direct to total anaerobic corrosion*

Whereas CMIC by sulfide leads to FeS as the only product (Eq. 4), EMIC leads in addition to non-sulfidic ferrous iron which tends to precipitate as carbonate (Eq. 5). Hence, the ratio of FeS to total Fe(II) in a crust should, in principle, allow to calculate the contribution of EMIC to MIC (EMIC and CMIC), the total corrosion due to the activity of SRB in the environment. Still, this is a rather formal treatment that does not consider higher levels of complexity, e.g. secondary (simultaneous or subsequent) reaction of sulfide from organotrophic SRB not only with the metal, but also with ferrous carbonate from EMIC ($Fe^0 \Leftarrow H_2S, HS^- \Rightarrow FeCO_3$), thus increasing the proportion of FeS ($FeCO_3 + H_2S \rightarrow FeS + HCO_3^- + H^+$). Hence, EMIC could be the real (yet 'masked') cause of corrosion even if all ferrous iron in the crust is present as FeS.

The contribution (mol/mol) of EMIC to MIC is formally expressed as the quotient of the amount of iron corroded by EMIC, $n_{FeEMIC}$, to the amount corroded by MIC, $n_{FeMIC}$, the latter being identical with the measurable ferrous iron formed, $n_{Fe(II)}$, so that

$$q_{EMIC} = \frac{n_{FeEMIC}}{n_{FeMIC}} = \frac{n_{FeEMIC}}{n_{Fe(II)}}. \quad (S35)$$

An expression must be found which besides $n_{Fe(II)}$ includes the other measurable amount, $n_{FeS}$, but no longer the not directly obvious $n_{FeEMIC}$. This is achieved by including four other equations. Two of these are equations (7) and (9),

$$n_{FeEMIC} = n_{FeCatab} + n_{FeAnab} \quad (S36)$$

and

$$q_{Anab} = \frac{n_{FeAnab}}{n_{FeEMIC}}, \quad (S37)$$

respectively. Equation (8) cannot be applied, because MIC includes also organic electron donors in addition to $Fe^0$ for sulfate reduction, so that $n_{FeCatab} < 4\,n_{SR}$. Furthermore, the characteristic product of EMIC is non-sulfidic iron, its amount being designated $n_{FeNonS}$. This is ¾ of the amount oxidized by the catabolism (Eq. 5) plus the amount resulting from biosynthesis, i.e.

$$n_{FeNonS} = \tfrac{3}{4}\,n_{FeCatab} + n_{FeAnab}. \qquad (S38)$$

Finally, the amount $n_{FeNonS}$ is iron totally formed by MIC (EMIC + CMIC), $n_{Fe(II)}$, less sulfidic iron, $n_{FeS}$, so that

$$n_{FeNonS} = n_{Fe(II)} - n_{FeS}. \qquad (S39)$$

For convenience, we arrange the coefficients and 'parameters' ($q_{EMIC}$, $q_{Anab}$) of equations (S35)–(S39) [order of rows below] by $n_{Fe(II)}$, $n_{FeS}$, $n_{FeEMIC}$, $n_{FeCatab}$, $n_{FeAnab}$, $n_{FeNonS}$ as

$$\left(\begin{array}{cccccc|c} q_{EMIC} & 0 & -1 & 0 & 0 & 0 & 0 \\ 0 & 0 & 1 & -1 & -1 & 0 & 0 \\ 0 & 0 & q_{Anab} & 0 & -1 & 0 & 0 \\ 0 & 0 & 0 & \tfrac{3}{4} & 1 & -1 & 0 \\ -1 & 1 & 0 & 0 & 0 & 1 & 0 \end{array}\right). \qquad (S40)$$

Because the two measurable 'variable' amounts, $n_{Fe(II)}$ and $n_{FeS}$, are finally of interest, the matrix is converted to

$$\left(\begin{array}{cccccc|c} [q_{EMIC}(3+q_{Anab})-4] & 4 & 0 & 0 & 0 & 0 & 0 \end{array}\right). \qquad (S41)$$

Hence,

$$[q_{EMIC}(3 + q_{Anab})]\,n_{Fe(II)} - 4\,n_{Fe(II)} + 4\,n_{FeS} = 0 \qquad (S42)$$

or

$$q_{EMIC} = \frac{4\,(1 - n_{FeS}/n_{Fe(II)})}{3 + q_{Anab}}. \qquad (S43)$$

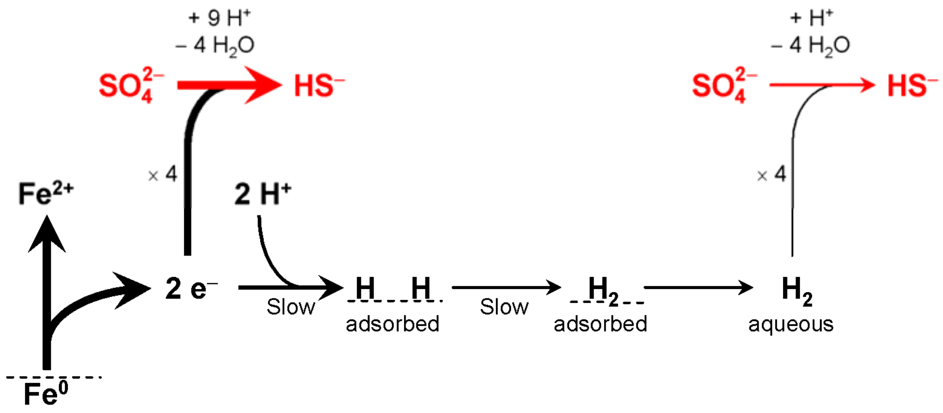


**Fig. S1.** Kinetic aspects of the abiotic reaction of iron in circumneutral water, and direct (lithotrophic) iron corrosion by SRB. Availability of $H^+$-ions at the metal surface and combination of adsorbed H-atoms to adsorbed $H_2$ are assumed to be rate-controlling steps ('bottle necks'), thus also controlling liberation of $H_2$ into water (Bockris and Reddy, 1970; Hamann *et al.*, 2007). $H_2$ consumption by SRB behind the bottle neck is therefore unlikely to promote iron dissolution. Direct consumption of electrons can oxidize the iron much faster. Thickness of arrows symbolizes speed. The net reaction is always
$4\ Fe^0 + SO_4^{2-} + 4\ H_2O \rightarrow FeS + 3\ Fe^{2+} + 8\ OH^-$.

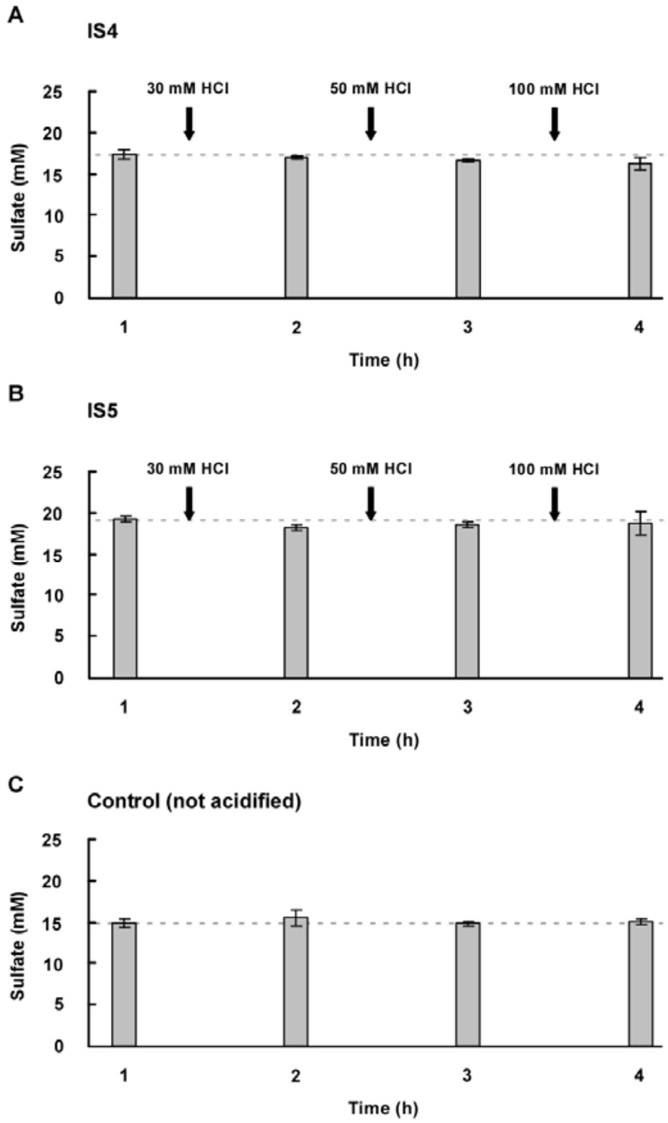


**Fig. S2.** Excluding disappearance of sulfate due to co-precipitation in the corrosion crust. Grown cultures were step-wise acidified with HCl until formed corrosion products were completely dissolved. Sulfate concentration of medium did not increase.
**A**. Culture of strain IS4.
**B**. Culture of strain IS5.
**C**. Control culture of strain IS4 which was not acidified.

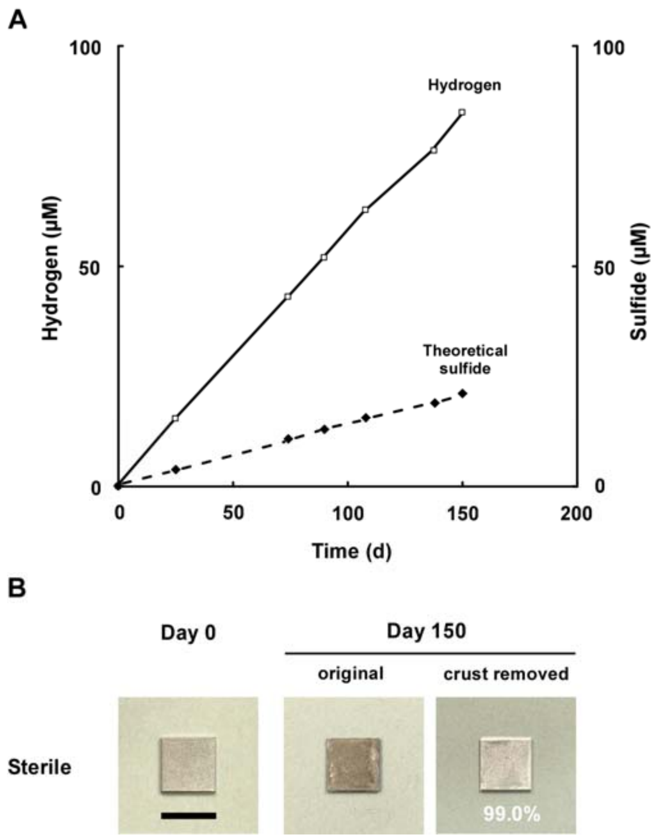


**Fig. S3.** Abiotic anaerobic iron corrosion in sterile synthetic seawater medium.
**A**. Production of 'cathodic' hydrogen by reduction of $H^+$ ions (Fig. S1), and sulfide that could be formed by $H_2$ utilization by SRB ($4\ H_2 + SO_4^{2-} + 2\ H^+ \rightarrow H_2S + 4\ H_2O$).
**B**. Original iron specimen (day 0), specimen with precipitate after 5 months (original) and after removal of precipitate (using HCl-hexamine).

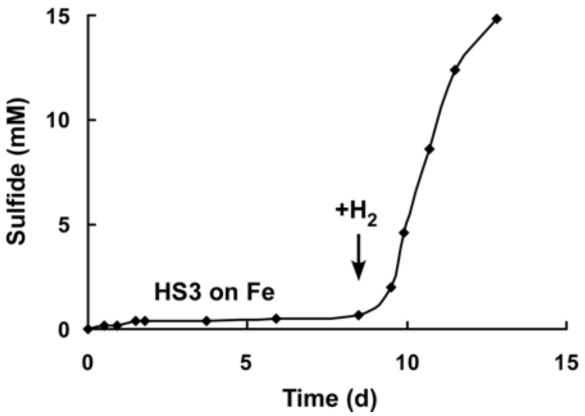


**Fig. S4.** Insensitivity of non-corrosive control strain HS3 towards $Fe^{2+}$. Addition of $H_2$ to the culture including iron granules leads to rapid sulfide production (measured as sulfate loss).

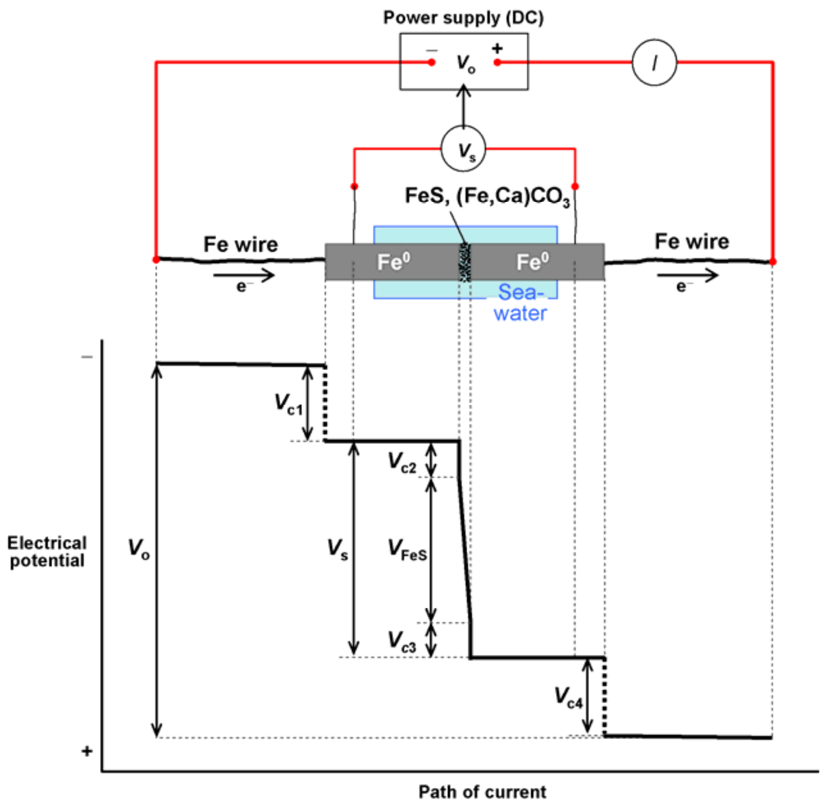


**Fig. S5.** Electro-technical scheme with approximate voltage drops of the split-coupon incubation device for conductance measurement of the biogenic crust formed on corroding iron. The device circumvents interference by the noticeable contact resistance between the iron wire and the iron coupon inside the incubated bottle (Fig. 3A). The plot in the lower part depicts the voltage drop along current flow. The outer voltage ($V_o$) is supplied and adjusted such that the voltage across the split ($V_s$) is kept at 0.20 V while the current ($I$) is being measured. The adjusted low voltage for measurement avoids electrolysis. Measurement of $V_s$ is carried out with a high-resistance voltmeter. $V_{c1}$ and $V_{c4}$ are the voltage drops due to contact resistance between the iron wire and the iron coupon (around 1 Ω), and $V_{c2}$ and $V_{c3}$ the arbitrarily assumed voltage drops due to the contact resistance between iron and the sulfidic crust. Voltage drop along the iron wire and the iron coupons is negligible (resistance by two and four orders or magnitude lower, respectively, than resistance of wire-coupon contact and the crust).

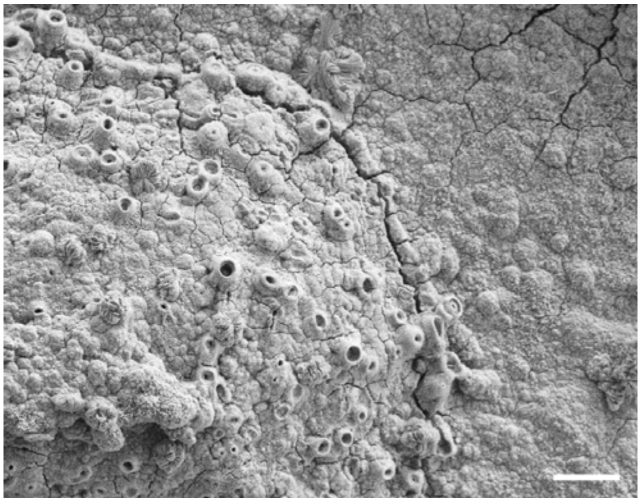

**Fig. S6.** Pustule (elevated precipitate) with early stage of micro-chimney formation above an anodic site in a culture of strain IS4 after three months of incubation. Bar, 50 µm.

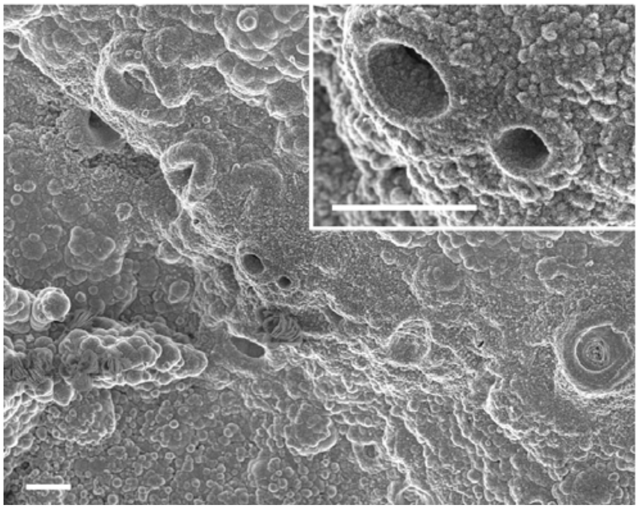

**Fig. S7.** Early stage of micro-chimney formation above an anodic area in a culture of strain IS4 after three months of incubation. Bar, 10 µm.

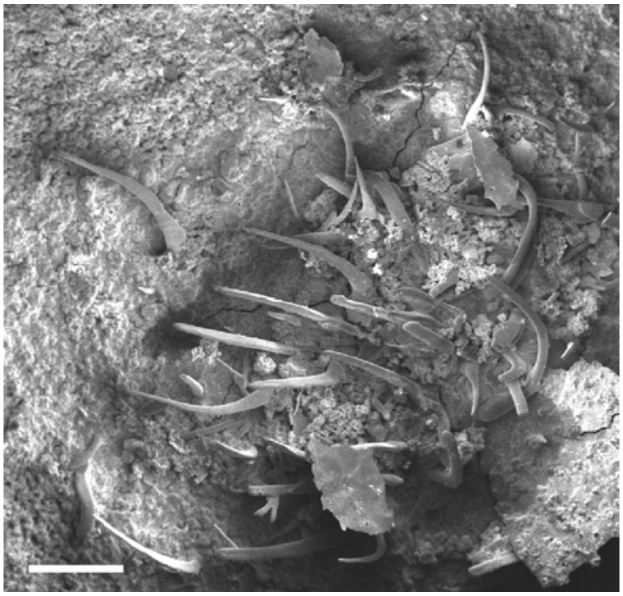

**Fig. S8.** Late stage of micro-chimney formation in a culture of strain IS4 after six months of incubation. Bar, 200 µm.

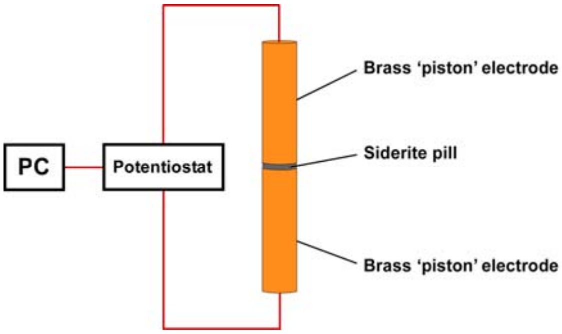


**Fig. S9.** Piston electrode set-up for measurement of conductivity of a compressed siderite mineral pill.

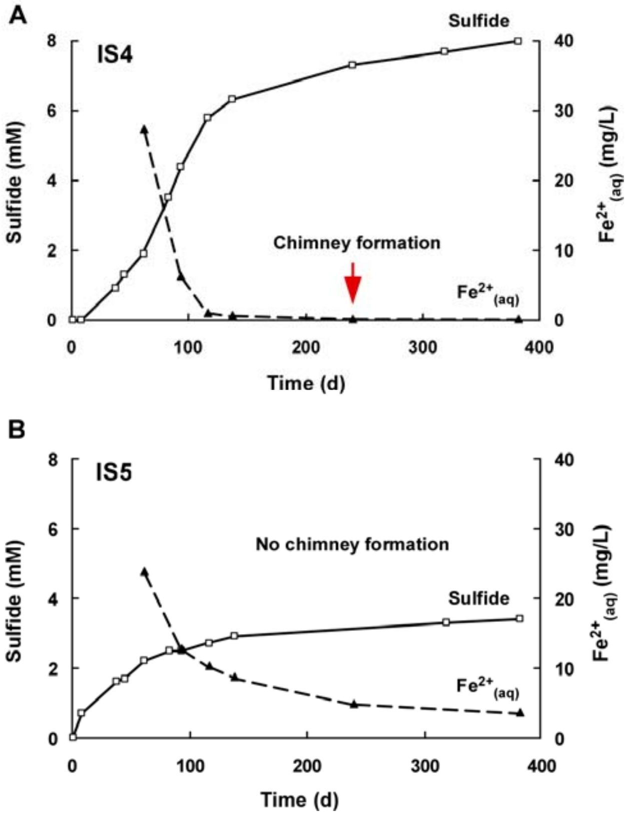


**Fig. S10.** Sulfide production (determined as sulfate consumption) and decrease of dissolved ferrous iron due to carbonate precipitation in long-term incubations of corrosive SRB. Strain IS4 (**A**) which was more alkali-tolerant than strain IS5 (**B**) grew up to higher *pH* [*pH* increase due to equation (5)] thus promoting precipitation according to $Fe^{2+} + HO^{-} + HCO_3^{-} \rightarrow FeCO_3 + H_2O$. This favored formation of micro-chimneys (Fig. 5C). Six cultures of each strain were incubated in parallel and sacrificed at different time points for SEM analysis (Fig. 4, Figs S6 to 8). Formation of crater- and chimney-like structures in cultures of strain IS4 coincided with the drop of [$Fe^{2+}_{(aq)}$] below detection limit (0.2 mg/l). The initial *pH* was 7.3. Strain IS4 reached *pH* ≈ 9. Activity of strain IS5 ceased at *pH* ≈ 8.

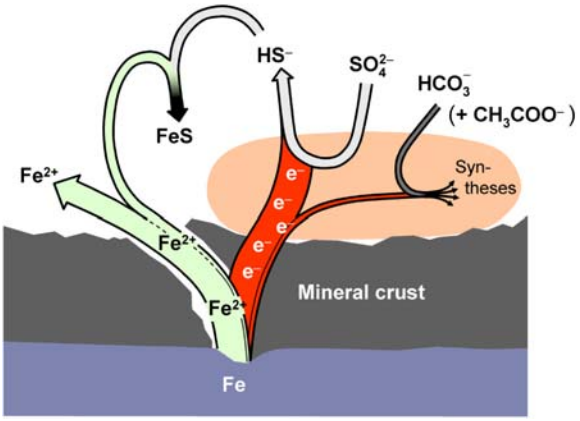


**Fig. S11.** Electron flow from metallic iron into the catabolism and anabolism.

**Table S1.** Compilation of corrosion rates recorded for (anoxic) natural and engineered environments, and for laboratory cultures of sulfate-reducing bacteria.

| Location | Type of environment | Corrosion rate (mm $yr^{-1}$)[a] | Method[b] | Reference |
|---|---|---|---|---|
| Bohai Bay, China | Marine sediment | **0.03 – 0.09** | Weight loss | Li (2009) |
| Draugen Oil Field, Norwegian North Sea | Produced water re-injection system; untreated produced water | **0.12** | Electrochemical[c] | Vik *et al.* (2007) |
| Tonnenlegerbucht, German North Sea | Marine anoxic sediment | **0.12 – 0.28** | Weight loss | This study |
| South Korea | Gas transmission pipelines; disbonded coating | **0.33 – 0.47** | Pit measurement | Li *et al.* (2000) |
| Gullfaks Oil Field, Norwegian North Sea | Water injection system; biocide treatment | **0.09 – 0.75** | Weight loss | Boedtker *et al.* (2008) |
| **Culture** | **Type of energy source, medium[d]** | **Corrosion rate (mm $yr^{-1}$)[e]** | **Method** | **Reference** |
| *Desulfovibrio sp.* | Lactate-based, freshwater | **0.007** | Weight loss | Hardy and Brown (1984) |
| *Desulfovibrio vulgaris*, Woolwich | Lactate-based, freshwater | **0.024** | Weight loss | Gaylarde (1992) |
| *Desulfovibrio desulfuricans*, New Jersey | Lactate-based, marine | **0.094** | Weight loss | Beech *et al.* (1994) |
| *Desulfovibrio desulfuricans* | Lactate-based, brackish | **0.285** | Weight loss | Bell and Lim (1981) |
| SRB bioreactor | Lactate-based, brackish | **0.408** | Weight loss | Hubert *et al.* (2005) |
| *Desulfovibrio vulgaris*, Hildenborough | $H_2$, freshwater[f] | **0.500** | Electrochemical[g] | Pankhania *et al.* (1986) |
| Sterile artificial seawater | $Fe^0$ lithotrophy, marine | **0.010** | Weight loss | This study |
| *Desulfopila inferna* | $Fe^0$ lithotrophy, marine | **0.012** | Weight loss | This study |
| *'Desulfopila corrodens'*, strain IS4 | $Fe^0$ lithotrophy, marine | **0.311** | Weight loss | This study |
| *'Desulfovibrio ferrophilus'*, strain IS5 | $Fe^0$ lithotrophy, marine | **0.710** | Weight loss | This study |

**a.** Corrosion rate in natural and engineered environments. The recorded range is given. Corrosion must not necessarily be influenced by SRB in these systems.
**b.** Method of corrosion rate determination. Weight loss is considered the most accurate technique.
**c.** A linear polarisation resistance (LPR) probe was used.
**d.** Energy source for SRB metabolism is indicated. All cultures contained iron or mild steel. '$Fe^0$ lithotrophy' indicates that metallic iron was the only available source of reducing equivalents. Salt content of media (freshwater, brackish, marine) is also designated.
**e**. Corrosion rate in cultures of SRB. The maximal reported value is given. The large range of corrosion rates for SRB with external energy source is attributed to the sometimes protective properties of formed FeS films (see text).
**f.** $H_2$ from previous polarisation experiment was available in these cultures.
**g.** Calculated from slope of cathodic polarisation curve by overvoltage-intercept method.

**Table S2.** Vitamins in used media.

| Vitamin | Concentration (µg $l^{-1}$) |
|---|---|
| 4-Aminobenzoic acid | 40 |
| D(+)-Biotin | 10 |
| Nicotinic acid | 100 |
| Ca-D(+)-Pantothenate | 50 |
| Pyridoxine hydrochloride | 150 |
| Folic acid | 40 |
| Liponic acid | 15 |
| Thiamine hydrochloride | 100 |
| Riboflavin | 25 |
| Cyanocobalamin | 50 |

**Table S3.** Conductivity values measured in an incubation device with split coupon with corrosive cultures of strains IS4 and IS5, and with sterile artificial seawater. Iron is provided as the sole source of electrons.

| | Slot bridged by sulphidic crust [a] | Slot bridged by seawater (control) [b] |
|---|---|---|
| **Supplied outer voltage ($V_o$)** | 0.2 – 0.4 V | 0.2 – 0.3 V |
| **Voltage at split coupon ($V_s$)** | 0.20 V | 0.20 V |
| **Measured current** | 0.14 – 0.31 A | $0.3 - 0.7 \cdot 10^{-4}$ A |
| **Conductance** | 0.72 – 1.56 S | $1.5 - 3.5 \cdot 10^{-4}$ S |
| **Conductivity** | 27.4 – 43.5 $S\ m^{-1}$ | $0.5 - 1.7 \cdot 10^{-2}\ S\ m^{-1}$ |
| **Resistance** | 0.64 – 1.39 Ω | $2.9 - 6.7 \cdot 10^{3}\ \Omega$ |
| **Resistivity** | $2.3 - 3.7 \cdot 10^{-2}\ \Omega m$ | $0.6 - 2.1 \cdot 10^{2}\ \Omega m$ |

**a.** Values refer to data range obtained from 4 biological replicates (3 cultures of strain IS4 and one culture of strain IS5);
**b.** Values refer to data range obtained from 3 sterile incubations.

**Table S4.** Electrical conductivity of selected substances.

| Material | Conductivity ($S\ m^{-1}$) | Reference |
|---|---|---|
| Iron, 99.98% pure | $1.1 \cdot 10^{7}$ | a |
| Steel, plain | $5.6 \cdot 10^{6}$ | b |
| Graphite | $1.5 \cdot 10^{6}$ | b |
| Steel, stainless | $1.4 \cdot 10^{6}$ | b |
| Troilite (FeS) | $1.0 \cdot 10^{1} - 1.0 \cdot 10^{6}$ | Pearce *et al.* (2006) |
| Pyrrhotite ($Fe_{1-X}S$), mineral | $2.0 \cdot 10^{4} - 1.0 \cdot 10^{5}$ | Parasnis (1956) |
| Pyrrhotite ($Fe_{1-X}S$), ore | $1.0 \cdot 10^{3} - 1.0 \cdot 10^{5}$ | Parasnis (1956) |
| Magnetite ($Fe_3O_4$) | $1.0 \cdot 10^{4} - 1.0 \cdot 10^{5}$ | Schwertmann & Cornell (2003) |
| Pyrite ($FeS_2$), mineral | $2.0 \cdot 10^{1} - 2.0 \cdot 10^{4}$ | Parasnis (1956) |
| Pyrite ($FeS_2$), ore | $1.0 \cdot 10^{-1} - 1.0 \cdot 10^{4}$ | Parasnis (1956) |
| **SRB corrosion crust** | $\mathbf{2.7 \cdot 10^{1} - 6.4 \cdot 10^{1}}$ | **This study** |
| Germanium | $2.2 \cdot 10^{0}$ | b |
| *G. sulfurreducens* biofilm | $0.5 \cdot 10^{0}$ | Malvenkar et al. (2011) |
| Silicon | $1.6 \cdot 10^{-3}$ | b |
| **Siderite mineral** | $\mathbf{1.2 \cdot 10^{-7}}$ | **This study** |
| Goethite (FeOOH) | approx. $1.0 \cdot 10^{-7}$ | Schwertmann & Cornell (2003) |
| Siderite ($FeCO_3$) | $1.2 \cdot 10^{-10}$ | Schön (1996) |
| Calcite ($CaCO_3$) | $2.0 \cdot 10^{-13} - 1.1 \cdot 10^{-14}$ | Schön (1996) |

**a.** CRC Handbook of Chemistry and Physics.
**b.** www.physics.info/electric-resistance/

## References

Atkins, P., and De Paula, J. (2006) *Physical Chemistry*. Oxford: Oxford University Press.

Beech, I.B., Cheung, C.W.S., Chan, C.S.P., Hill, M.A., Franco, R., and Lino, A.R. (1994) Study of parameters implicated in the biodeterioration of mild steel in the presence of different species of sulfate-reducing bacteria. *Int Biodet Biodegr* **34**: 289–303.

Beech, I.B., and Sunner, I.A. (2007) Sulphate-reducing bacteria and their role in corrosion of ferrous materials. In *Sulphate-reducing bacteria: environmental and engineered systems*. Barton, L.L., and Hamilton, W.A. (eds). Cambridge: Cambridge University Press, pp. 459–482.

Bell, R.G., and Lim, C.K. (1981) Corrosion of mild and stainless steel by four tropical *Desulfovibrio desulfuricans* strains. *Can J Microbiol* **27**: 242–245.

Bockris, J.O.M., and Reddy, A.K.N. (1970) *Modern Electrochemistry*. New York: Plenum.

Bødtker, G., Thorstenson, T., Lillebø, B.L.P., Thorbjørnsen, B.E., Ulvøen, R.H., Sunde, E., and Torsvik, T. (2008) The effect of long-term nitrate treatment on SRB activity, corrosion rate and bacterial community composition in offshore water injection systems. *J Ind Microbiol Biotechnol* **35**: 1625–1636.

Dean, J.A. (1992) *Lange's Handbook of Chemistry*. New York: McGraw-Hill.

Dinh, H.T., Kuever, J., Mußmann, M., Hassel, A.W., Stratmann, M., and Widdel, F. (2004) Iron corrosion by novel anaerobic microorganisms. *Nature* **427**: 829–832.

Gaylarde, C.C. (1992) Sulfate-reducing bacteria which do not induce accelerated corrosion. *Int Biodet Biodegr* **30**: 331–338.

Hamann, C.H., Hamnett, A., and Vielstich, W. (2007) *Electrochemistry*, 2nd edn. Weinheim: Wiley.

Hardy, J.A., and Bown, J.L. (1984) The corrosion of mild steel by biogenic sulfide films exposed to air. *Corrosion* **40**: 650–654.

Hubert, C., Nemati, M., Jenneman, G., and Voordouw, G. (2005) Corrosion risk associated with microbial souring control using nitrate or nitrite. *Appl Microbiol Biot* **68**: 272-282.

Jack, T.R. (2002) Biological corrosion failures. In *ASM Handbook Volume 11: Failure Analysis and Prevention*. Shipley, R.J., and Becker, W.T. (eds). Materials Park: ASM International, pp. 881–898.

Koch, G.H., Brongers, M.P.H., Thompson, N.G., Virmani, Y.P., and Payer, J.H. (2001) Corrosion cost and preventive strategies in the United States. In: CC Technologies Laboratories NACE International.

Lee, W., Lewandowski, Z., Nielsen, P.H., and Hamilton, W.A. (1995) Role of sulfate-reducing bacteria in corrosion of mild-steel - a review. *Biofouling* **8**: 165–194.

Li, Y.T. (2009) Corrosion behaviour of steel in beach soil along Bohai Bay. *Corrosion Eng Sci Tech* **44**: 91–95.

Li, S., Kim, Y., Jeon, K., and Kho, Y. (2000) Microbiologically influenced corrosion of underground pipelines under the disbonded coatings. *Met Mater* **6**: 281–286.

Malvankar, N.S., Vargas, M., Nevin, K.P., Franks, A.E., Leang, C., Kim, B.C. et al. (2011) Tunable metallic-like conductivity in microbial nanowire networks. *Nat Nanotech* **6**: 573–579.

Pankhania, I.P., Moosavi, A.N., and Hamilton, W.A. (1986) Utilization of cathodic hydrogen by *Desulfovibrio vulgaris* (Hildenborough). *J Gen Microbiol* **132**: 3357–3365.

Parasnis, D.S. (1956) The electrical resistivity of some sulphide and oxide minerals and their ores. *Geophys Propect* **4**: 249–279.

Patrick, W.A., and Thompson, W.E. (1952) Standard electrode potential of the iron-ferrous ion couple at 25°. *J Am Chem Soc* **75**: 1184−1187.

Pearce, C.I., Pattrick, R.A.D., and Vaughan, D.J. (2006) Electrical and magnetic properties of sulfides. *Rev Mineral Geochem* **61**: 127–180.

Randall, M., and Frandsen, M. (1932) The standard electrode potential of iron and the activity coefficient of ferrous chloride. *J Am Chem Soc* **54**: 47−54.

Revie, R.W. (2011) *Uhlig's Corrosion Handbook*. New York: Wiley.

Rickard, D., and Luther, G.W. (2007) Chemistry of iron sulfides. *Chemical Reviews* **107**: 514−562.

Schwertmann, U., and Cornell, R.M. (2003) *The Iron Oxides*. Weinheim: Wiley.

Schön, J.H. (1996) Electrical properties of rocks. In *Physical properties of rocks: Fundamentals & principles of petrophysics*. Schön, J.H. (ed). Oxford: Pergamon Press, p. 379.

Vik, E.A., Janbu, A.O., Garshol, F., Henninge, L.B., Engebretsen, S., Kuijvenhoven, C. et al. (2007) Nitrate-based souring mitigation of produced water - side effects and challenges from the Draugen produced water re-injection pilot, paper SPE 106178. In *SPE International Symposium on Oilfield Chemistry*. Houston.

## Symbols used in equations (SI units; and other, convenient units)

| | |
|---|---|
| $a$ | Area ($m^2$; $cm^2$) |
| $d$ | Dinstance, length (m) |
| $E_{SR}$ | Equilibrium redox potential of sulfate reduction (V) |
| $E_{FeDiss}$ | Equilibrium redox potential of iron dissolution (V) |
| $E°_{pH7}$, $E°'$ | Equilibrium redox potential at pH 7, but otherwise standard conditions (V) |
| $K_{eq}$, $K_a$ | Equilibrium constant |
| $F$ | Faraday constant (C $mol^{-1}$) |
| $\phi$ | Operational redox potential (V) |
| $I$ | Electrical current (A) |
| $i$ | Current density (A $m^{-2}$) |
| $i_{corr}$ | Corrosion current density (A $m^{-2}$) |
| $M_a$ | Atomic mass (kg $mol^{-1}$) |
| $M_A$ | Atomic mass of substance A (kg $mol^{-1}$; g $mol^{-1}$) |
| $m_A$ | Mass of substance A (kg) |
| $m_{Bio}$ | Biomass (kg; g) |
| $m_{Min}$ | Mass of minerals in corrosion crust (kg) |
| $m_{AeCO3}$ | Mass of alkaline earth (Mg, Ca) carbonates (kg) |
| $n$ | Amount (mol) |
| $n_A$ | Amount of substance A (mol) |
| $n_{FeAnab}$ | Amount of iron oxidized anabolically for biosynthesis (mol) |
| $n_{FeCatab}$ | Amount of iron oxidized catabolically by sulfate reduction (mol) |
| $n_{FeEMIC}$ | Amount of $Fe^0$ oxidized by EMIC (mol) |
| $n_{FeNonS}$ | Amount of non-sulfidic iron (mol) |
| $n_{Fe(II)}$ | Amount of total ferrous iron formed, equivalent with $n_{\Delta Fe(0)}$ (mol) |
| $n_{\Delta Fe(0)}$ | Amount of metallic iron lost by anaerobic oxidation, equivalent with $n_{Fe(II)}$ (mol) |
| $n_{FeS}$ | Amount of sulphidic iron (mol) |
| $n_e$ | Number of electrons released per metal atom |
| $q$ | Quotient, molar (mol $mol^{-1}$) |
| $q_{EMIC}$ | Quotient iron oxidized by EMIC per total iron oxidized by MIC (mol $mol^{-1}$) |
| $q_{Anab}$ | Quotient iron oxidized for biosynthesis per total iron oxidized by EMIC (mol $mol^{-1}$) |
| $q^{m}_{Bio}$ | Quotient biomass per total corrosion crusts (kg $kg^{-1}$) |
| $\rho$ | Density (kg $m^{-3}$) |
| $\sigma$ | Electrical conductivity (S $m^{-1}$) |
| $t$ | Time (s; yr) |
| $\theta$ | Metal thickness (m; mm) |
| $v^{m}_{corr}$ | Rate of metal mass loss (kg $s^{-1}$; mg $yr^{-1}$) |
| $v^{n}_{corr}$ | Rate of metal amount loss (mol $s^{-1}$; mmol $yr^{-1}$) |
| $v^{\theta}_{corr}$ | Rate of metal thickness loss (m $s^{-1}$; mm $yr^{-1}$) |
| $V$ | Here: voltage (V); otherwise volume |
| $Y_{Anab}$ | Growth yield, cell mass per $Fe^0$ oxidized with sulfate (kg $mol^{-1}$; g $mol^{-1}$) |
