# Supplementary figures and images for: Marine sulfate-reducing bacteria cause serious corrosion of iron under electroconductive biogenic mineral crust
Source: Environ Microbiol. 2012 Jul;14(7):1772–87. doi: 10.1111/j.1462-2920.2012.02778.x (PMC3429863; doi:10.1111/j.1462-2920.2012.02778.x)

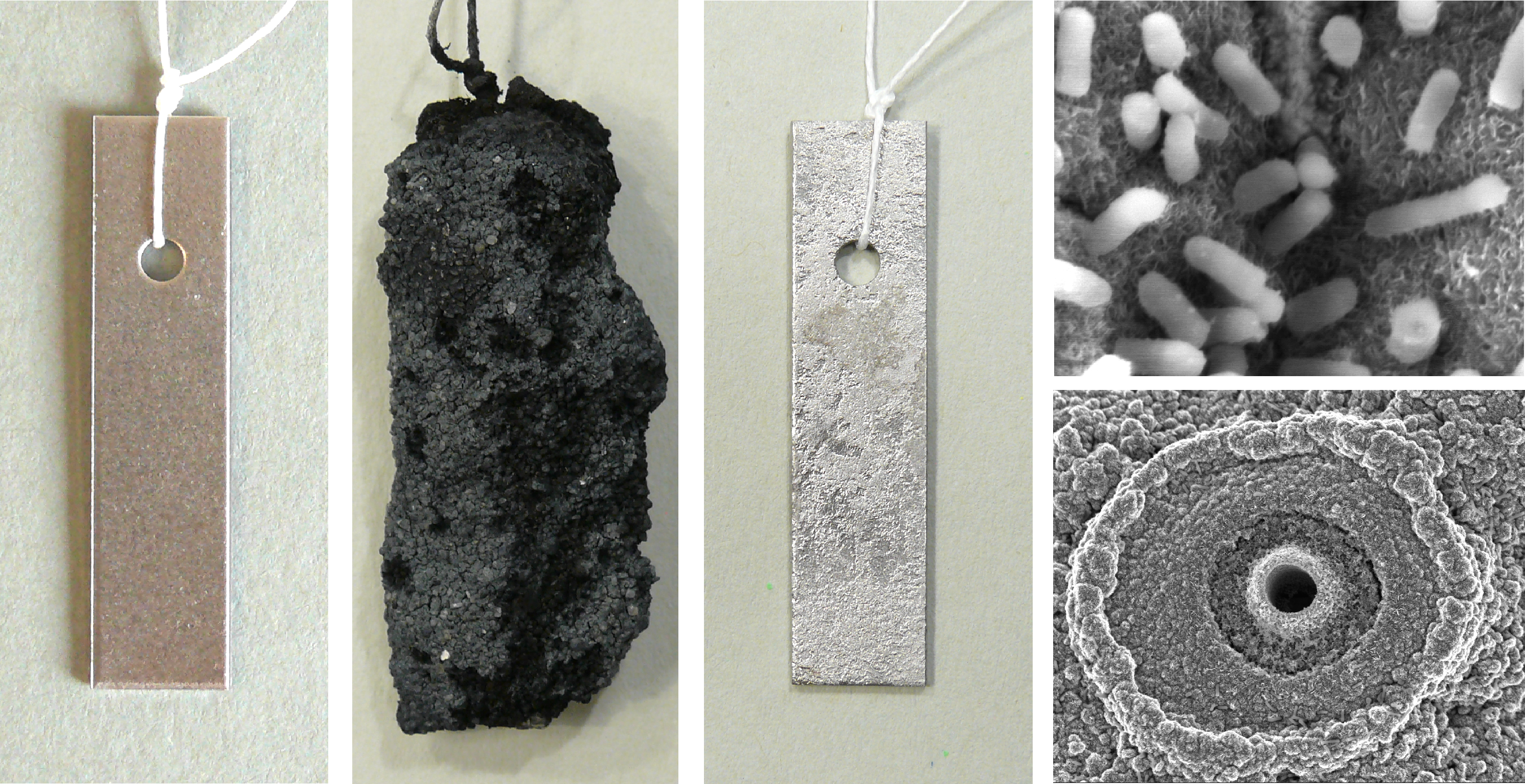

Supplement: Supplementary file 17 [file emi0014-1772-SD17.tif]

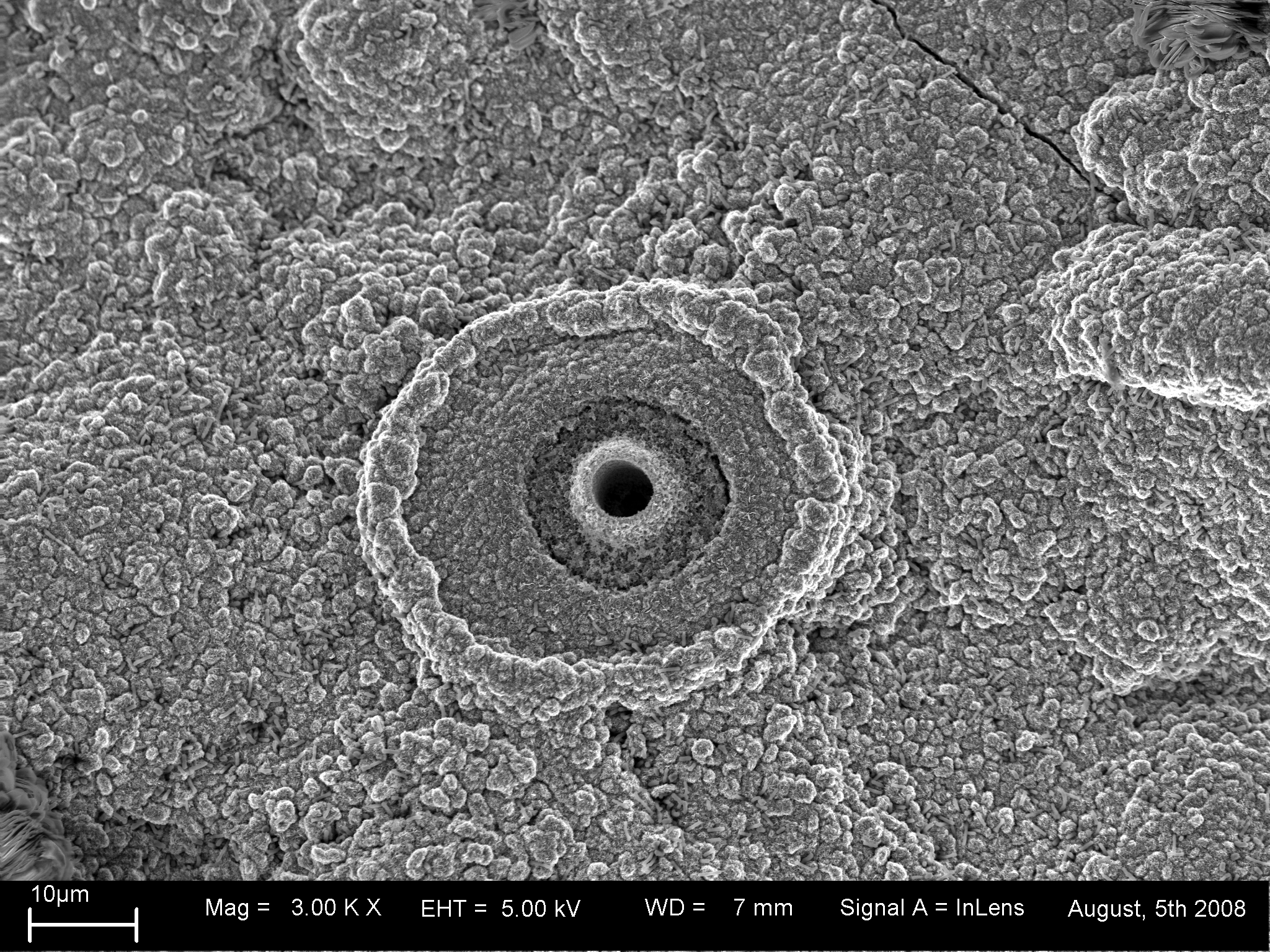

Supplement: Supplementary file 18 [file emi0014-1772-SD18.tif]
